# Phosphorylation of FAM134C by CK2 controls starvation-induced ER-phagy

**DOI:** 10.1126/sciadv.abo1215

**Published:** 2022-08-31

**Authors:** Giorgia Di Lorenzo, Francescopaolo Iavarone, Marianna Maddaluno, Ana Belén Plata-Gómez, Simone Aureli, Camila Paz Quezada Meza, Laura Cinque, Alessandro Palma, Alessio Reggio, Carmine Cirillo, Francesca Sacco, Alexandra Stolz, Gennaro Napolitano, Oriano Marin, Lorenzo A. Pinna, Maria Ruzzene, Vittorio Limongelli, Alejo Efeyan, Paolo Grumati, Carmine Settembre

**Affiliations:** ^1^ Telethon Institute of Genetics and Medicine (TIGEM), Pozzuoli, Italy.; ^2^ Metabolism and Cell Signaling Laboratory, Spanish National Cancer Research Centre (CNIO), Madrid, Spain.; ^3^ Università della Svizzera italiana (USI), Faculty of Biomedical Sciences, Euler Institute, Lugano, Switzerland.; ^4^ Department of Biomedical Sciences, University of Padova, Padova, Italy.; ^5^ Department of Clinical Medicine and Surgery, Federico II University, Naples, Italy.; ^6^ Department of Biology, University of Rome “Tor Vergata”, Rome, Italy.; ^7^ Institute of Biochemistry II, Faculty of Medicine, Goethe University, Frankfurt am Main, Germany.; ^8^ Buchmann Institute for Molecular Life Sciences (BMLS), Goethe University, Frankfurt am Main, Germany.; ^9^ Department of Translational Medicine, Federico II University, Naples, Italy.; ^10^ CNR Neuroscience Institute, Padova, Italy.; ^11^ Department of Pharmacy, Federico II University, Naples, Italy.

## Abstract

Selective degradation of the endoplasmic reticulum (ER) via autophagy (ER-phagy) is initiated by ER-phagy receptors, which facilitate the incorporation of ER fragments into autophagosomes. FAM134 reticulon family proteins (FAM134A, FAM134B, and FAM134C) are ER-phagy receptors with structural similarities and nonredundant functions. Whether they respond differentially to the stimulation of ER-phagy is unknown. Here, we describe an activation mechanism unique to FAM134C during starvation. In fed conditions, FAM134C is phosphorylated by casein kinase 2 (CK2) at critical residues flanking the LIR domain. Phosphorylation of these residues negatively affects binding affinity to the autophagy proteins LC3. During starvation, mTORC1 inhibition limits FAM134C phosphorylation by CK2, hence promoting receptor activation and ER-phagy. Using a novel tool to study ER-phagy in vivo and FAM134C knockout mice, we demonstrated the physiological relevance of FAM134C phosphorylation during starvation-induced ER-phagy in liver lipid metabolism. These data provide a mechanistic insight into ER-phagy regulation and an example of autophagy selectivity during starvation.

## Teaser

The ER-phagy receptor FAM134C is regulated during starvation by CK2-mediated phosphorylation.

## INTRODUCTION

(Macro)autophagy begins with the sequestration of cytosolic substrates in autophagic vesicles that subsequently fuse with lysosomes where cargo degradation occurs (*1*). Nutrient starvation represents the most potent physiological inducer of autophagy. Starvation-induced autophagy is mainly controlled by mechanistic target of rapamycin complex 1 (mTORC1) that phosphorylates members of the autophagy initiation complex ULK1 and ATG13 and the transcription factors TFEB and TFE3 (*2*–*6*). The mechanisms controlling autophagy substrate capture during starvation-induced autophagy are not well defined, and this type of autophagy is considered a bulk process.

The selection of substrates during autophagy can be mediated by autophagy receptors, which are able to bind cytosolic cargoes facilitating their sequestration into forming autophagosomes. Autophagy receptors associate with the LC3 and GABARAP proteins on autophagic membranes through the LC3-interating region (LIR) and GABARAP-interacting motif, respectively (*7*, *8*). Selective autophagy cargo includes the endoplasmic reticulum (ER) (ER-phagy), mitochondria (mitophagy), lipid droplets (lipophagy), and several other cellular components.

ER-phagy is emerging as a critical quality control mechanism to preserve ER homeostasis (*9*–*14*). FAM134B was described as the first mammalian autophagy receptor that controls the degradation of the ER (*15*), and its activity can be modulated at both transcriptional and posttranslational levels (*16*–*19*). Subsequently, additional ER membrane proteins have been characterized as ER-phagy receptors (TEX264, SEC62, RTN3L, CCPG1, and ATL3) (*20*–*25*). Recently, we further expanded this knowledge by characterizing the two FAM134B paralogs, FAM134A and FAM134C (*26*). FAM134 proteins represent the first ER-phagy receptor family. Despite the strong homology in the LIR region and the presence of a reticulon homology domain in all of them, the FAM134 family members have several unique characteristics. For example, FAM134B overexpression is sufficient to promote ER-phagy (*17*), while FAM134A and FAM134C appear to be inactive in steady-state conditions (*26*). Whether functional changes in the structure and/or the interactome profiles of FAM134A and FAM134C are needed for their activation is currently unknown.

In this study, we identified a mechanism of FAM134C activation that contributes to ER-phagy induction during starvation. FAM134C activation is controlled by casein kinase 2 (CK2)–dependent phosphorylation at critical and unique residues that are proximal to the LIR motif. This phosphorylation event modulates the interaction between FAM134C and LC3 proteins, and it is modulated during starvation in an mTORC1-dependent manner. We showed the physiological relevance of FAM134C phosphorylation in vivo and, using in vivo tools to study ER-phagy, an altered FAM134C mediated activation of ER-phagy in mice with aberrant mTORC1 signaling.

## RESULTS

### Regulation of FAM134 proteins by starvation and mTORC1 inhibition

**Fig. 1.**
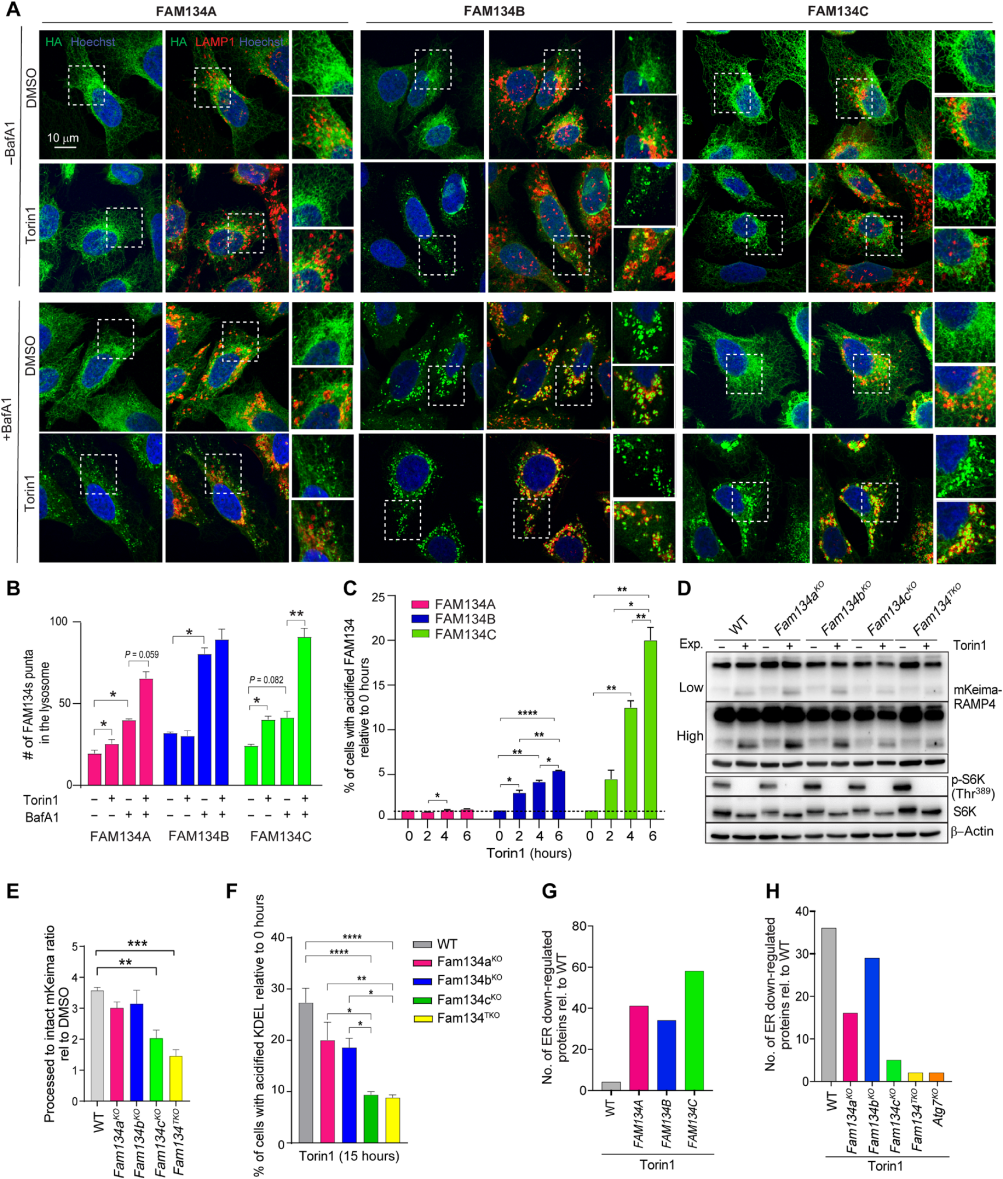
Regulation of FAM134 protein by mTORC1 inhibition. (**A**) Representative images of immunofluorescence staining for HA, LAMP1, and nuclei in HA-FAM134s–expressing U2OS cells cultured with BafA1 (200 nM; 4 hours) and/or Torin1 (150 nM; 8 hours), where indicated. Scale bar, 10 μm. (**B**) Quantification of HA puncta into LAMP1-positive vesicles. Mean ± SEM. *N* = 3 biological replicates. *n* = 15 cells per experiment. Two-way analysis of variance (ANOVA) and Tukey’s multiple comparison test: *P < 0.05 and **P < 0.005. (**C**) Fluorescence-activated cell sorting (FACS) analysis in human embryonic kidney (HEK) 293T cells expressing ssRFP-GFP-FAM134s. Torin1 (150 nM) was added for the indicated time points. Quantification of red fluorescence shift relative to untreated (0 hours). Mean ± SEM. *N* = 4 biological replicates. Two-way ANOVA and Tukey’s multiple comparison test: ****P < 0.0001, **P < 0.005, and *P < 0.05. (**D**) Representative Western blot analysis in RCS cells expressing mKeima-RAMP4 treated with Torin1 (150 nM; 15 hours). β-Actin was used as a loading control. (**E**) Quantification of mKeima/mKeima-Ramp4 ratio relative to dimethyl sulfoxide (DMSO)–treated cells. Mean ± SEM. *N* = 4 biological replicates. One-way ANOVA and Tukey’s multiple comparison test: **P < 0.005 and ***P < 0.0005. (**F**) FACS analysis in RCS cells expressing ssRFP-GFP-KDEL treated with Torin1 (150 nM; 15 hours). Quantification of red fluorescence shift relative to untreated. Mean ± SEM. *N* = 6 biological replicates. One-way ANOVA and Tukey’s multiple comparison test: ****P < 0.0001, **P < 0.005, and *P < 0.05. (**G** and **H**) Proteome of U2OS (G) and RCS cells (H) untreated or treated with Torin1 (150 nM; 12 hours). Bar graphs show the number of ER proteins down-regulated by Torin1 (150 nM; 12 hours) in control [wild type (WT)] and HA-FAM134–overexpressing U2OS cells (G) and in RCS with indicated genotypes (H). *N* = 3 biological replicates. Adjusted *P* value < 0.05

To study the response of the FAM134 proteins to starvation and mTOR inhibition, we used U2OS cells that express doxycycline-inducible hemagglutinin (HA)-FAM134A, HA-FAM134B, and HA-FAM134C at comparable levels (fig. S1A). We analyzed FAM134 lysosomal flux before and after treatment with the mTOR inhibitor Torin1 and in the presence of bafilomycin A1 (BafA1), an inhibitor of lysosomal degradation. The addition of BafA1 to cells for 4 hours resulted in a massive accumulation of FAM134B, but not of FAM134A and FAM134C, within LAMP1-positive vesicles, thus indicating that FAM134B lysosomal flux was higher compared to the other family members (Fig. 1, A and B), consistent with previous observations (*26*).

The addition of Torin1 stimulated FAM134C and FAM134A lysosomal puncta formation, with FAM134C showing a more robust response (Fig. 1, A and B). Conversely, Torin1 did not further increase FAM134B lysosomal puncta number, in the presence and absence of BafA1 (Fig. 1, A and B).

Next, we generated human embryonic kidney (HEK) 293 cells stably expressing FAM134 proteins tagged with red fluorescent protein–enhanced green fluorescent protein (RFP-EGFP) to measure FAM134 delivery to lysosomes in response to a time course of Torin1 treatment by fluorescence-activated cell sorting (FACS) analysis (Fig. 1C). The acidic environment inside the lysosome efficiently quenches the fluorescent signal of EGFP but not of RFP, and the fluorescent shift can be measured by FACS. We observed that FAM134C was the most responsive to Torin1 administration, showing about 20-fold increased acidification upon 6 hours of Torin1 administration. Differently from what we observed in U2OS cells, Torin1 enhanced FAM134B lysosomal delivery in HEK293 cells, although at much lower levels compared to FAM134C.

To assess the relative contributions of the FAM134 proteins during Torin1-induced ER-phagy, we generated RCS cells lacking either *Fam134a*, *Fam134b*, *Fam134c*, or all three combined (*Fam134*^TKO^) (fig. S1B) and expressing the mKeima reporter fused to the ER membrane protein RAMP4. The mKeima protein is unaffected by lysosomal proteases, and the appearance of a proteolytically cleaved mKeima from RAMP4 can be used to measure lysosomal delivery of RAMP4-decorated ER fragments (*20*, *27*). We observed that *Fam134c^KO^* and *Fam134^TKO^* showed a significant stronger impairment of Torin1-induced ER-phagy compared with the other genotypes, as measured by the levels of processed mKeima (Fig. 1, D and E). Similarly, FACS analysis in Torin1-treated control and Fam134 knockout (KO) cells expressing the fluorescent ER-phagy reporter SS-RFP-GFP-KDEL demonstrated reduction of ER-phagy flux in all *Fam134* KO cells, with *Fam134c^KO^* and *Fam134^TKO^* having the strongest effects (Fig. 1F). To gain insights into the functional relevance of these observations, we performed full proteome mass spectrometry (MS) analysis on U2OS and RCS cells that overexpress and lack FAM134 proteins, respectively. In both U2OS and RCS control cells, Torin1 treatment significantly down-regulates several ER proteins in an ER-phagy–dependent fashion, because this effect was blunted in *Atg7^KO^* and *Fam134^TKO^* cells (Fig. 1, G and H; fig. S2; and table S5). We found that the overexpression of HA-FAM134C increased the number of ER proteins down-regulated by Torin1 stimulation to a higher extent compared with HA-FAM134B or HA-FAM134A overexpression (Fig. 1G and table S4). Furthermore, *Fam134c^KO^* caused the strongest inhibition of Torin1-induced ER protein degradation compared with *Fam134b* and *Fam134a* deletions (Fig. 1H, fig. S2, and table S5). Collectively, gain- and loss-of-function experiments performed in different cell types and using diverse approaches uncovered an unexpected prominent role of FAM134C, over other family members, during mTORC1-dependent starvation-induced ER-phagy. These data prompted us to search for a possible mechanism of FAM134C activation during starvation and mTORC1 inhibition.

### FAM134C phosphorylation at critical residues controls its activation during starvation

**Fig. 2.**
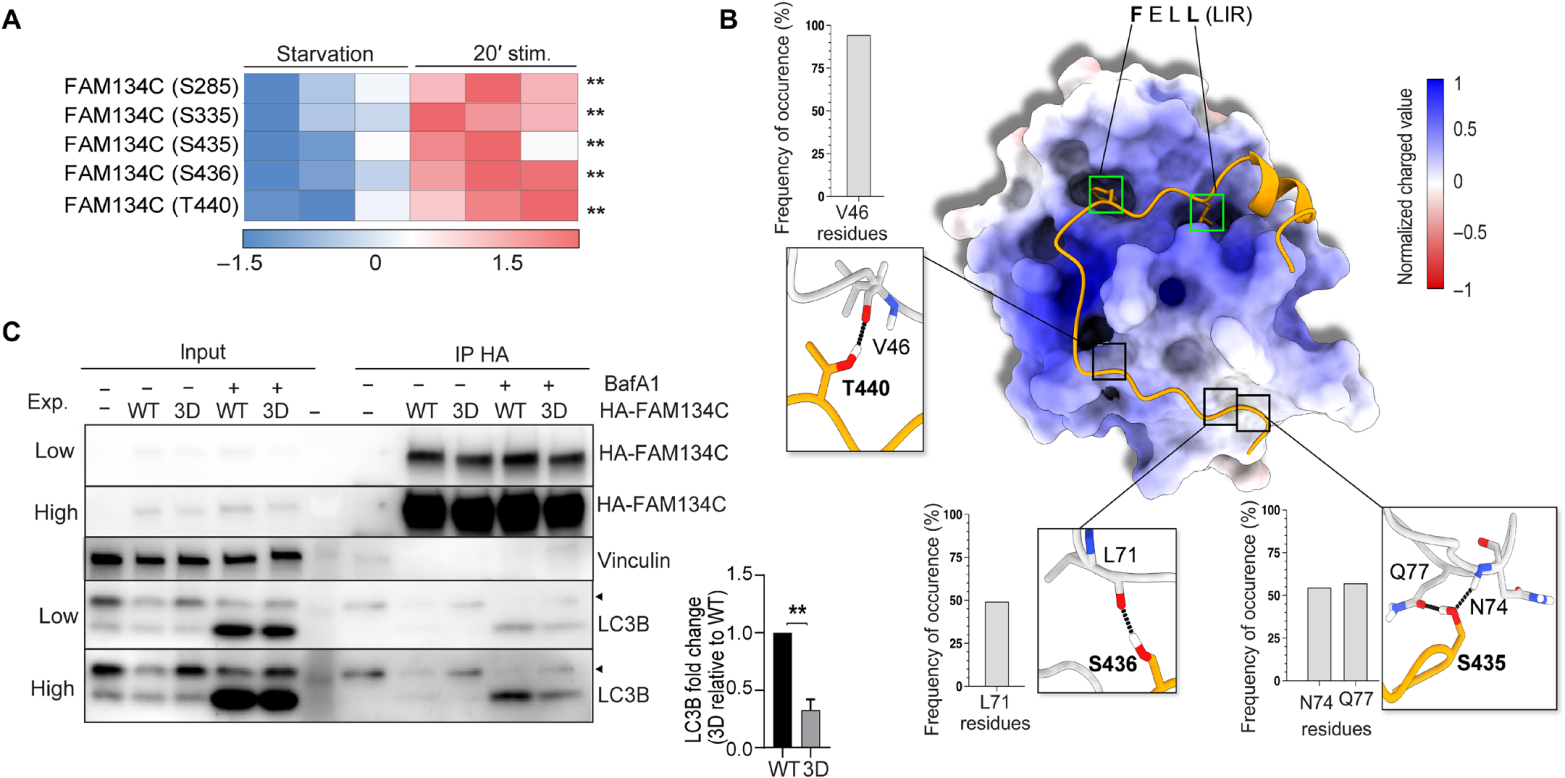
Phosphorylation of FAM134C controls its affinity for LC3B. (**A**) Heatmap of the phosphorylation levels of FAM134C at indicated residues in U2OS cells cultured in starved (HBSS medium) versus stimulated (amino acids + 100 nM insulin) medium. Heatmap is color-coded according to the intensity level of the phosphorylated site. *N* = 3 biological replicates. FDR: **P < 0.005. (**B**) Structural analysis of the FAM134C_P434-P460/LC3B complex. Projection of the electrostatic potential map of LC3B onto the structure of the centroid associated with the most populated cluster family. The insets display the interactions established by Ser^435^, Ser^436^, and Thr^440^ of FAM134C with LC3B and their frequency of occurrence in the PT-MD principle conformational state. LC3B was represented through its solvent-accessible surface, colored according to the local electrostatic potential map values following the color gradient on the right side. FAM134C_P434-P460 was represented in ribbon and colored in orange. Green boxes highlight the first (F) and the fourth (L) amino acids of the FAM134C LIR domain. (**C**) Western blot analysis showing immunoprecipitation (IP) experiment of HA-FAM134C-WT and HA-FAM134C-3D overexpressed in U2OS cells. Low and high refers to different exposures of the membrane. BafA1 (200 nM) was supplied where indicated. Arrowheads indicate a nonspecific band. On the right, bar graph shows quantification of LC3B binding of FAM134C-3D relative to WT in the presence of BafA1. Mean ± SEM. *N* = 3 biological replicates. Student’s paired *t* test: **P < 0.005.

**Fig. 3.**
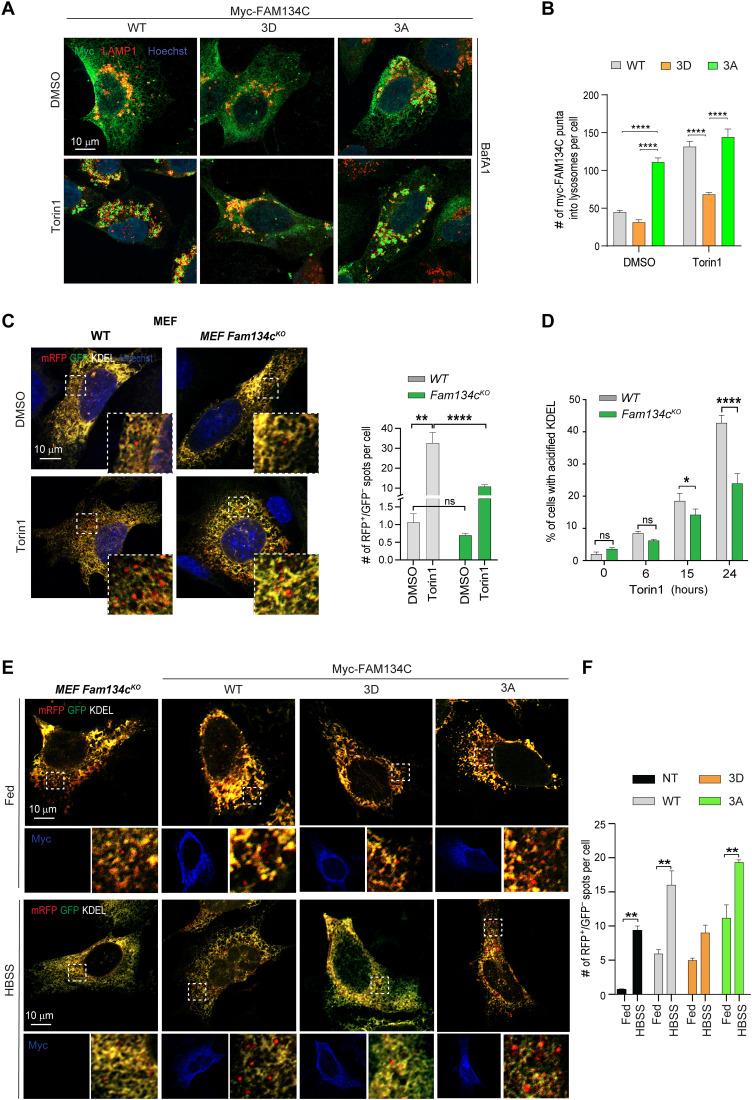
FAM134C activity is regulated by phosphorylation. (**A**) Representative immunofluorescence staining of Myc, LAMP1, and nuclei in U2OS cells transfected with WT-, 3D-, and 3A-Myc-FAM134C. Cells were treated for 2 hours with BafA1 (100 nM), and where indicated, Torin1 (250 nM) was added for 6 hours. Scale bar, 10 μm. (**B**) Quantification of Myc-FAM134C lysosomal puncta per cell. Bar graph shows mean ± SEM. *N* = 3 biological replicates. *n* = 15 cells per experiment. Two-way ANOVA and Tukey’s multiple comparison test: ****P < 0.0001. (**C**) Representative immunofluorescence staining for RFP, GFP, and nuclei in WT and *Fam134c^KO^* MEF cells, stably expressing the ER-phagy reporter ssRFP-GFP-KDEL, after Torin1 treatment (150 nM; 24 hours). Scale bar, 10 μm. Quantification shows the number of red-only positive puncta (ssRFP^+^) per cell. Mean ± SEM. *N* = 3 biological replicates. *n* = 15 cells per experiment. Two-way ANOVA and Sidak’s and Tukey’s multiple comparison test: ***P < 0.0005 and **P < 0.005. ns, not significant. (**D**) Quantification of red fluorescent shift performed by FACS analysis in WT and *Fam134c^KO^* MEF cells, stably expressing ssRFP-GFP-KDEL, treated with Torin1 (150 nM) or DMSO at the indicated time points. *N* = 3 biological replicates. Two-way ANOVA and Sidak’s and Tukey’s multiple comparison test: ****P < 0.0001 and *P < 0.05. (**E**) Representative images of *Fam134c^KO^* MEFs cells expressing ssRFP-GFP-KDEL and transfected with WT, 3D, or 3A mutant FAM134C proteins. Cells were kept in complete medium or starved for 8 hours in HBSS. Scale bars, 10 μm. (**F**) Quantification of red-only puncta (RFP^+^/GFP^−^) average per cell. Bar graph shows mean ± SEM. *N* = 3 biological replicates. *n* = 15 cells per experiment. Two-way ANOVA and Tukey’s multiple comparison test: **P < 0.005 and *P < 0.05.

The lysosomal delivery of HA-FAM134C was potently induced in response to serum, amino acids, or their combined deprivation [Hanks’ balanced salt solution (HBSS) medium] in U2OS cells (fig. S3A). Phosphoproteomic analysis comparing starved (HBSS) with nutrient- and insulin-rich medium (full medium) U2OS cells expressing HA-FAM134C identified specific residues located in the C terminus of FAM134C (S285, S335, S435, S436, and T440) significantly phosphorylated in full versus starved medium conditions (Fig. 2A). The analysis of a published phosphoproteome dataset of starved versus insulin-stimulated Hepa-1-6 cell lines (*28*) confirmed that endogenous FAM134C was significantly more phosphorylated at S436 and T440 in insulin-stimulated conditions (fig. S3B). The S435, S436, and T440 residues are in the cytosolic C-terminal region of the protein in proximity (−12, −11, and −7, respectively) of the LIR sequence that binds to LC3/GABARAP proteins (starting at F447). To elucidate the role played by these residues, we investigated the binding mode between FAM134C and LC3B. As no experimental structure was available, we first generated a homology model of the FAM134C binding interface encompassing the residues P434 to P460 (FAM134C_P434-P460 hereafter) (Fig. 2B and fig. S4, A to I). Molecular docking simulations and parallel-tempering molecular dynamics (PT-MD) calculations of the FAM134C/LC3B complex were performed to identify the interprotein contacts that stabilize the heterodimer structure. These methods are widely used to study molecular binding systems (*29*, *30*). The PT-MD calculations showed that all the residues S435, S436, and T440 engage in H-bond interactions with LC3B. FAM134C’s T440 and S435 form H-bonds with the backbone oxygens of LC3B V46 and L71, respectively, while FAM134C’s S435 engages in two H-bonds, one with the backbone oxygen of LC3B N74 and the other with the Q77 side chain (Fig. 2B). The full list of the FAM134C_P434-P460/LC3B interactions is reported in table S6. The obtained structure of the FAM134C_P434-P460/LC3B binding complex highlights the role played by S435, S436, and T440 in the formation and stability of the heterodimer, prompting us to further investigate the impact of the phosphorylation of these amino acids. To this end, we computed the electrostatic map of LC3B through the “Adaptive Poisson-Boltzmann Solver” software (*31*). Here, it can be noted that the region of LC3B interacting with FAM134C’s S435, S436, and T440 is relatively neutral in terms of charge, suggesting that phosphorylation of the indicated residues, increasing the local electron density, might lead to a less favorable interaction with LC3B. We also investigated how single and triple mutations S435D, S436D, and T440D in FAM134C affect the binding affinity to LC3B. The substitution of serine and threonine residues with aspartate allows assessing the result of charge modification in FAM134C, mimicking the effect of phosphorylation at these sites. Our results show that both the triple mutant (3D) and the single mutants S435D and S436D strongly decrease the FAM134C binding score to LC3B, while such effect is less pronounced in the T440D mutant (see fig. S4, J to L). On the other hand, the triple mutant resulting from the substitution of S435, S436, and T440 with alanine (3A), instead of aspartate, has a binding score like that of the wild type (WT). These results support the model that phosphorylation at S435, S436, and, at a less extent, T440 could affect the FAM134C binding to LC3B. To experimentally prove the functional role of FAM134C phosphorylation, we mutagenized S435, S436, and T440 residues into alanine [phosphomutant (3A)] or aspartic acid [phosphomimetic (3D)], and consistent with the in silico analysis, coimmunoprecipitation (co-IP) experiments showed a reduced interaction of 3D-FAM134C compared to FAM134C WT with endogenous LC3B (Fig. 2C). In unstimulated conditions, 3D-Myc-FAM134C showed a reticular localization like WT-Myc-FAM134C. However, unlike WT-Myc-FAM134C, the 3D-Myc-FAM134C protein was relocalized significantly less in LAMP1-positive vesicles in the presence of Torin1 and BafA1 (Fig. 3, A and B). Conversely, we observed that 3A-Myc-FAM134C formed multiple LAMP1-positive puncta even in the absence of Torin1 (Fig. 3, A and B). Similar data were obtained analyzing colocalization of WT-, 3A-, and 3D-Myc-FAM134C proteins with the autophagosome marker LC3 (fig. S5). These data suggest that phosphorylation of FAM134C at S435, S436, and T440 controls its activation during ER-phagy by regulating FAM134C affinity for LC3B. To corroborate these results, we reconstituted mouse embryonic fibroblast (MEF) *Fam134c^KO^* with WT-, 3D-, and 3A-Myc-FAM134C proteins (fig. S6). MEF *Fam134c^KO^* had no ER-phagy defects in basal conditions but showed impaired mTOR-dependent activation of ER-phagy (Fig. 3, C and D), which was rescued by reintroducing WT- or 3A-Myc-FAM134C but not 3D-Myc-FAM134C protein (Fig. 3, E and F).

### CK2 phosphorylates and controls lysosomal delivery of FAM134C

**Fig. 4.**
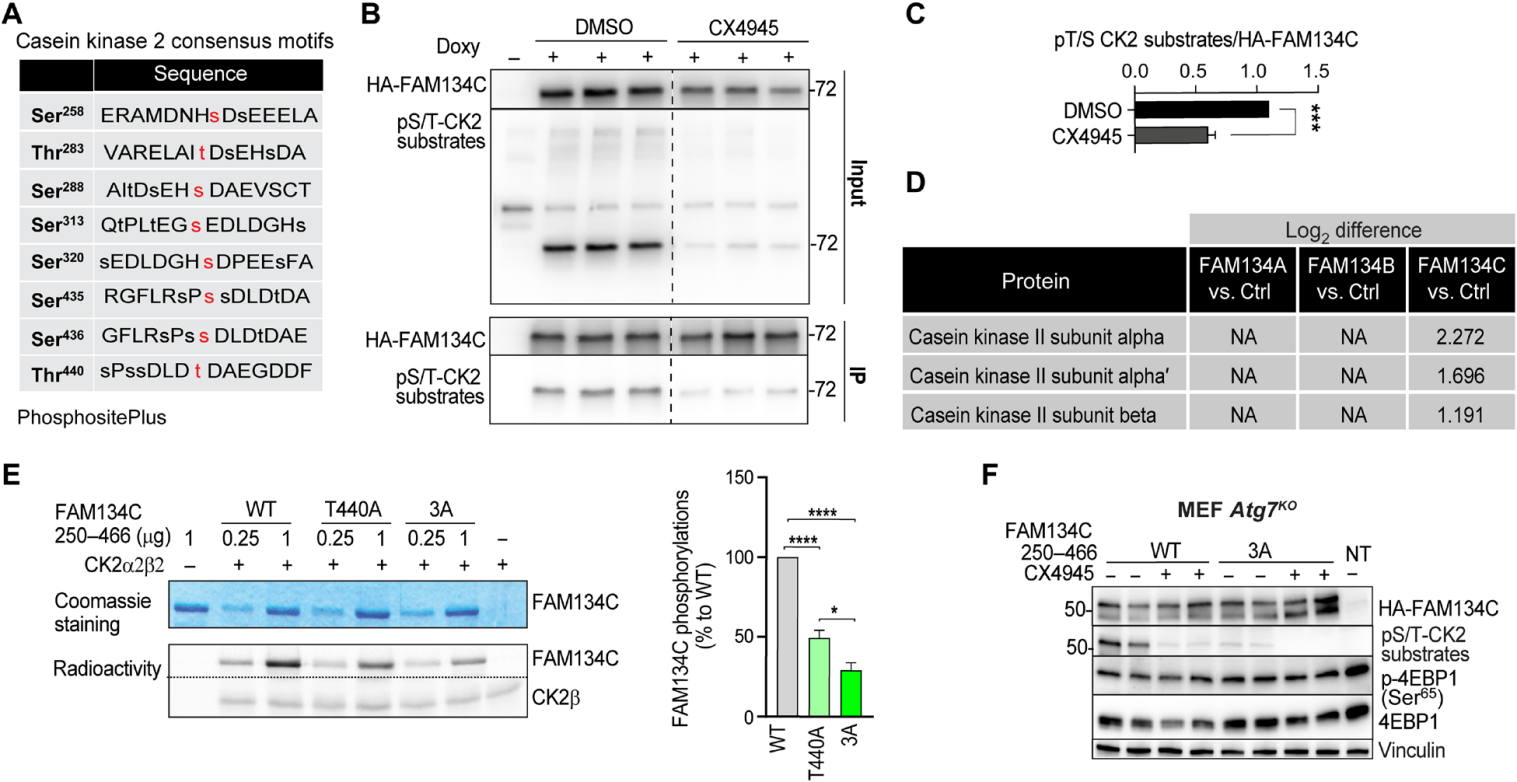
CK2 phosphorylates FAM134C. (**A**) List of putative residues with CK2 consensus at the C terminus of FAM134C (data obtained from the PhosphositePlus website). (**B**) U2OS cells expressing doxycycline-inducible HA-tagged FAM134C were subjected to IP. Inputs and immunoprecipitates were analyzed by immunoblotting using the indicated antibodies. (**C**) Bar graph shows quantification of phosphorylated/total HA-FAM134C ratio relative to (B). Mean ± SEM. *N* = 3 biological replicates. Student’s paired *t* test: ***P < 0.0005. CX4945: CK2 inhibitor (4 μM; 6 hours) and BafA1 (200 nM; 6 hours). (**D**) CK2 α and β subunit interaction with FAM134 proteins based on IP-MS interactome experiment from U2OS FLAG-HA-FAM134A, FLAG-HA-FAM134B, and FLAG-HA-FAM134C doxycycline-inducible cells. NA, not detected. Numeric values represent log_2_ difference. (**E**) In vitro CK2 phosphorylation radioactive assay. C-terminal FAM134C WT and phosphomutant (T440A and 3A) peptides and CK2β (control substrate) were incubated with CK2α in the presence of radioactive adenosine triphosphate (ATP). Substrate phosphorylation was detected by autoradiography. Equal amounts of proteins were verified by Coomassie staining. On the bottom, relative quantitation of the bands is performed by analysis with the CyclonePlus Storage Phosphor System (PerkinElmer); the unit amounts of each peptide are indicated, and activity is reported as % of that measured with 5 units of CK2α. Mean ± SEM. *N* = 3 biological replicates. One-way ANOVA and Tukey’s multiple comparison test: ****P < 0.0001 and *P < 0.05. (**F**) Western blot analysis of CK2-dependent phosphorylation of WT- and 3A-HA-FAM134C^250–466^ peptides transfected in *Atg7^KO^* MEFs untreated or treated with the CK2 inhibitor CX4945 (4 μM; 6 hours). Vinculin was used as loading control. NT, not transfected.

**Fig. 5.**
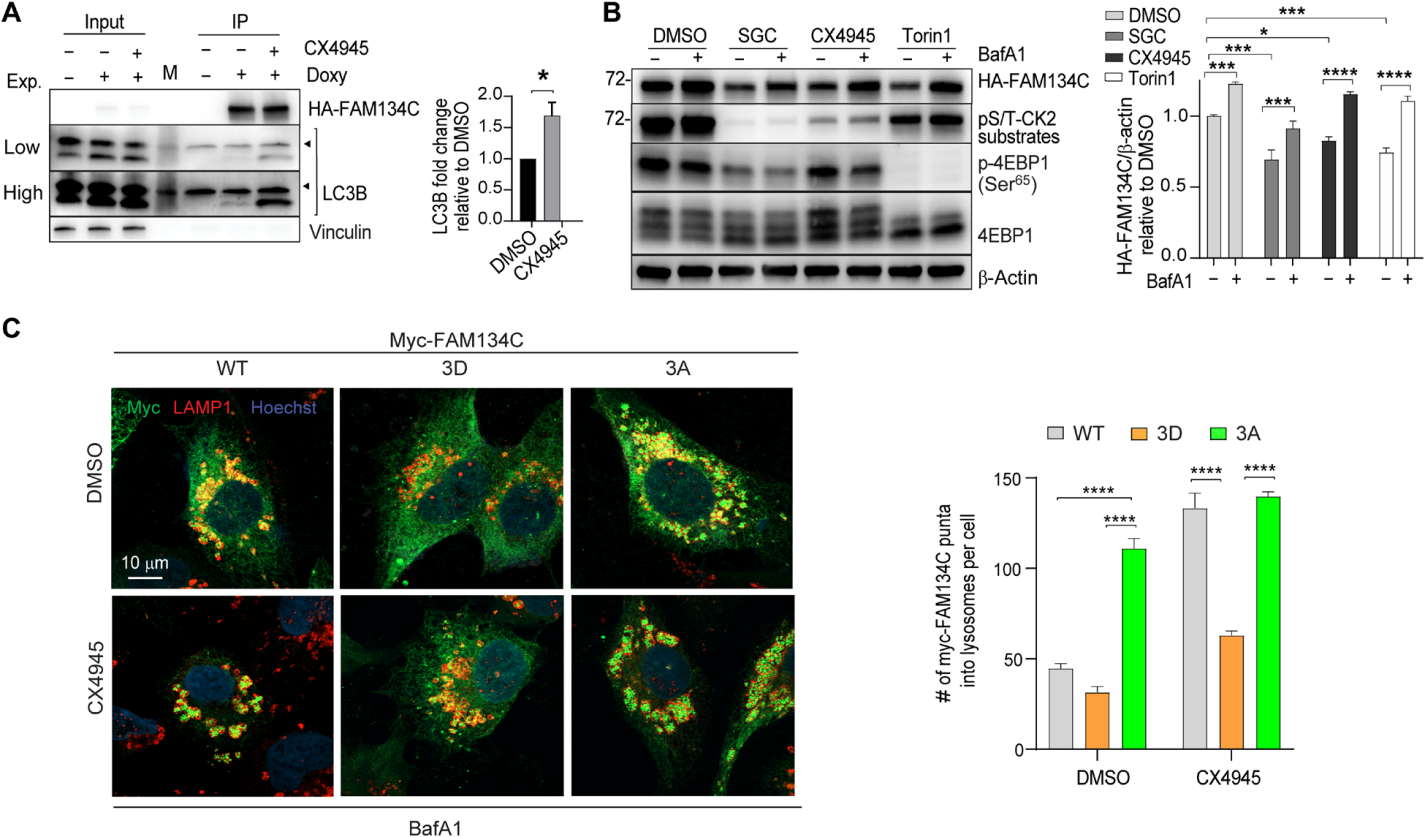
Regulation of FAM134C by CK2. (**A**) Representative Western blot analysis of IP experiments of doxycycline-inducible HA-FAM134C treated with CX4945 was indicated (4 μM; 4 hours). Vinculin was used as loading control for input. Arrowheads indicate a nonspecific band. On the right, the bar graph shows quantification of immunoprecipitated LC3B relative to untreated samples. Mean ± SEM. *N* = 3 biological replicates. Student’s paired *t* test: *P < 0.05. (**B**) Representative Western blot analysis of HA-FAM134C in U2OS cells overexpressing doxycycline-inducible HA-FAM134C treated with DMSO, Torin1 (250 nM; 6 hours), CK2 inhibitor CX4945 (4 μM; 6 hours), or CK2 inhibitor SGC-CK2-1 (50 nM; 6 hours) in the absence or presence of BafA1 (100 nM). β-Actin was used as a loading control. Bar graph shows quantification of HA-FAM134C/β-actin ratio relative to DMSO-treated samples. Mean ± SEM. *N* = 3 biological replicates. Sidak’s and Tukey’s multiple comparison test: ****P < 0.0001, ***P < 0.0005, and *P < 0.05. (**C**) Representative immunofluorescence staining of Myc (green), LAMP1 (red), and nuclei (blue) in U2OS cells transiently transfected with Myc-FAM134C-WT, Myc-FAM134C-3D, and Myc-FAM134C-3A. Cells were pretreated for 2 hours with BafA1 (100 nM), and then DMSO or CK2 inhibitor CX4945 (4 μM) was added for 6 hours. Scale bar, 10 μm. On the right, bar graph shows quantification of Myc-FAM134C in LAMP1-positive vesicles. Mean ± SEM. *N* = 3 biological replicates. *n* = 15 cells per experiment. Two-way ANOVA and Tukey’s multiple comparison test: ****P < 0.0001.

The FAM134C region containing residues S435, S436, and T440 shows the consensus binding sequence for protein kinase CK2, a constitutively active serine/threonine kinase whose holoenzyme is composed of two catalytic subunits (α and α^I^) and a dimer of regulatory β subunits (*32*, *33*). We analyzed an IP-MS experiment on phospho-Ser/Thr peptides using a CK2 substrate antibody (S*/T*D/EXD/E) to identify CK2 substrates and observed that S435, S436, and T440, as well as S258, T283, S288, S313, and S320, in FAM134C, are bona fide CK2 phosphorylation sites (PhosphoSitePlus database) (Fig. 4A) (*34*). Consistently, a commercially available phospho-CK2 substrate antibody detected immunoprecipitated FAM134C. Moreover, this detection was blunted by preincubating cells with the CK2 inhibitor CX4945 (Fig. 4, B and C). IP-MS of the FAM134 proteins demonstrated that FAM134C, but not FAM134A or FAM134B, interacts with CK2 α, α^I^, and β subunits (Fig. 4D and table S7). To formally demonstrate that FAM134C is a bona fide CK2 substrate, we generated WT and 3A mutant FAM134C fragments, encompassing the C-terminal cytosolic region of FAM134C (FAM134C^250–466^), where the phosphorylation occurs. An in vitro kinase assay showed that CK2 phosphorylates WT-FAM134C^250–466^ and that the phosphorylation efficiency was significantly reduced using the 3A-FAM134C^250–466^ variant (Fig. 4E). The residual phosphorylation observed incubating the 3A-FAM134C^250–466^ mutant with CK2 is consistent with the presence of additional CK2 sites in the FAM134C^250–466^ peptide (Fig. 4A). When transfected in cells, WT-FAM134C^250–466^, but not the 3A-FAM134C^250–466^ mutant, is recognized by the phospho-CK2 substrate antibody (Fig. 4F). This detection was abrogated by CX4945 pretreatment (Fig. 4F). Consistent with a phosphorylation-dependent mechanism of FAM134C regulation, the pharmacological inhibition of CK2 enhanced full-length FAM134C binding efficiency with endogenous LC3B (Fig. 5A) and promoted WT- but not 3D-Myc-FAM134C lysosomal localization and degradation (rescued by BafA1) (Fig. 5, B and C). Similar data were obtained using a different pharmacological inhibitor of CK2 (SGC-CK2-1) (Fig. 5B). Notably, CK2 inhibition had no effect on FAM134A and FAM134B delivery to lysosomes (fig. S7A). Together, these data suggest that FAM134C is a bona fide CK2 substrate and that CK2 controls FAM134C, but not FAM134A and FAM134B, activity.

CK2 inhibition failed to induce ER-phagy in U2OS cells expressing the ER-phagy reporter SS-RFP-GFP-KDEL. The Protein Atlas database (www.proteinatlas.org) indicates that *FAM134C* is highly expressed in pancreatic endocrine cells, collecting duct cells and alveolar cells. We confirmed these data comparing FAM134C transcript and protein levels between A549 (alveolar cells) and U2OS cell lines. Moreover, we tested ER-phagy in A549, stably expressing the ER-phagy reporter RFP-GFP-KDEL, and found that CK2 inhibition promoted ER-phagy in A549, but not in U2OS, cells (fig. S7, B to D). These data suggest that CK2 controls ER-phagy flux in cells expressing high levels of endogenous FAM134C.

### mTORC1 inhibition promotes FAM134C activation via CK2 regulation

**Fig. 6.**
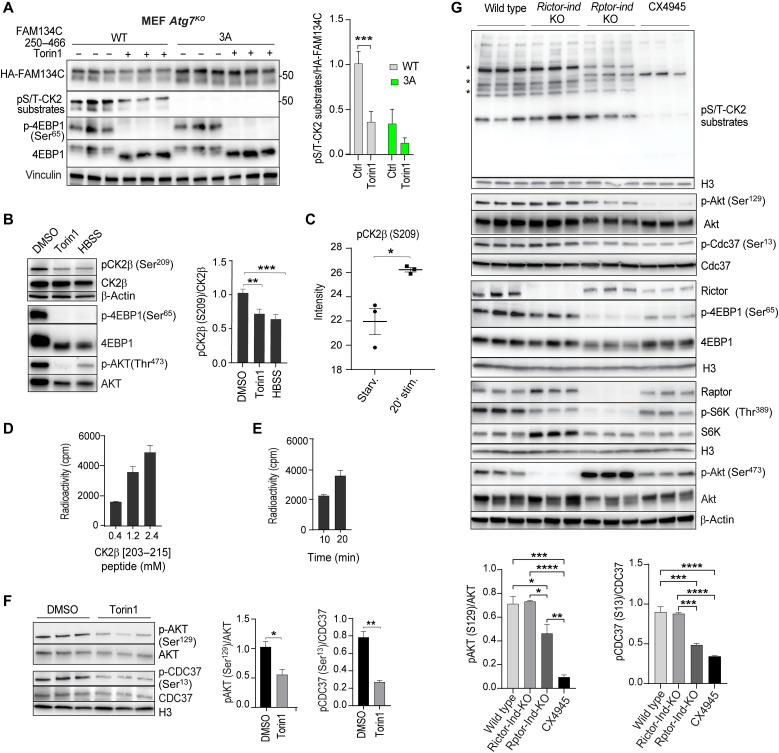
CK2-dependent phosphorylation of FAM134C is modulated by mTORC1. (**A**) Western blot analysis of *Atg7^KO^* MEF cells transfected with WT- or 3A-HA-FAM134C^250–466^ peptides and treated with Torin1 (1 μM; 6 hours). (**B**) Western blot analysis of U2OS cells transfected with GFP-CK2β plasmid treated with DMSO, Torin1 (1 μM), or HBSS (4 hours). Vinculin (A) or β-actin (B) was used as loading controls. Quantification (A and B) of phosphorylated/total protein ratio. *N* = 6 (A) and *N* = 4 (B) biological replicates. Mean ± SEM. Two-way ANOVA and Sidak’s multiple comparison test: **P < 0.005 and ***P < 0.0005. (**C**) Phosphorylation analysis of CK2β in U2OS cells. *N* = 3 biological replicates. Student’s paired *t* test: *P < 0.05. (**D** and **E**) Radioactive phosphorylation assays using (D) mTOR (200 ng; 20 min) and CK2β [203–215] peptide at the indicated concentrations and (E) mTOR (200 ng) and CK2β [203–215] peptide (1.2 mM) for the indicated times. Mean ± SEM. *N = 2*. (**F**) Western blot analysis of MEF cells treated with DMSO or Torin1 (1 μM; 6 hours). *N* = 3 biological replicates. H3 histone was used as loading control. Bar graphs show quantifications (mean ± SEM) of phosphorylated AKT and CDC37/total protein ratio. Student’s paired *t* test: *P < 0.05 and **P < 0.005 (**G**) Western blot analysis of WT, tamoxifen-inducible Raptor KO (*Rptor-indKO*), or Rictor KO (*Rictor-indKO*) MEFs. WT MEFs were treated with CX4945 (4 μM; 6 hours) as control. Asterisk (*) denotes bands changing intensity. *N* = 3 biological replicates. Bar graphs show quantifications (mean ± SEM) of phosphorylated AKT and CDC37/total protein ratio. Two-way ANOVA and Sidak’s multiple comparison test: *P < 0.05, **P < 0.005, ***P < 0.0005, and ****P < 0.0001.

Because starvation conditions induced FAM134C dephosphorylation at residues that are phosphorylated by CK2 (Figs. 2A and 4A and fig. S3B), we investigated whether FAM134C phosphorylation at S435, S436, and T440 was also modulated by mTOR inhibition. To prevent autophagy-mediated degradation of FAM134C upon Torin1 administration, we analyzed phosphorylation levels of WT-FAM134C^250–466^ and 3A-FAM134C^250–466^ fragments in RCS cells treated with BafA1 and in MEFs lacking the autophagy protein Atg7. We found that the phosphorylation levels of the WT-FAM134C^250–466^ peptide, but not of the 3A-FAM134C^250–466^ peptide, were significantly reduced by Torin1 treatment in both cell lines (Fig. 6A and fig. S8A), supporting the model that FAM134C phosphorylation can be modulated by mTOR inhibition.

We tested whether CK2 activity was influenced by starvation and mTOR inhibition. CK2 kinase assay on recombinant CK2 incubated with different concentrations of Torin1 demonstrated that CK2 is not directly inhibited by Torin1 (fig. S8B). In yeast, starvation and mTORC1 inhibition were reported to modulate the affinity of CK2 for its substrates through the phosphorylation of the CK2 regulatory subunit β1 at Ser^111^ (*35*). We found that the level of CK2β phosphorylation at Ser^209^, which is located in close proximity to the positive regulatory region domain of CK2β (*33*), was significantly reduced by both HBSS and Torin1 treatments (Fig. 6, B and C). Ser^209^ CK2β has a proline in +1 position, having a sequence that is compatible with the sites phosphorylated by mTOR. In vitro peptide phosphorylation assay using a commercial truncated form of mTOR kinase demonstrated that mTOR can phosphorylate a peptide reproducing the sequence of human CK2β [203–215] including Ser^209^ in a concentration- and time-dependent manner (Fig. 6, D and E) at levels that are similar to the known mTORC1 substrate Thr^389^ of S6K (fig. S8C). The overlay of literature-derived CK2 substrates obtained from PhosphoSitePlus (*34*) with our phosphoproteomic dataset reveals that the phosphorylation levels of 18 bona fide CK2 substrates were significantly different in stimulated versus starved conditions (fig. S8D). Torin1 treatment reduced the phosphorylation of AKT Ser^129^ (*36*) and CDC37 Ser^13^ (*37*), two well-established reporters of CK2 activity (Fig. 6F). In MEFs where mTORC1 and mTORC2 activity can be inhibited via tamoxifen-inducible deletion of Raptor or Rictor subunits, respectively (*Rptor/Rictor*-indKO MEFs) (*38*), we observed reduced phosphorylation levels of AKT Ser^129^ and CDC37 Ser^13^ in *Rptor*-indKO, but not in *Rictor*-indKO, MEFs (Fig. 6G). Furthermore, probing the phospho-CK2 substrate antibody on total cell lysates, we observed that deletion of *Rptor*, but not *Rictor*, altered the CK2 phosphorylation pattern (Fig. 6G). Although we cannot exclude that other cellular elements might interpose between mTORC1 and CK2 in vivo, collectively, these data suggest that CK2 activity on a subset of substrates can be modulated during starvation by mTORC1, providing a possible explanation for the mTORC1-mediated regulation of FAM134C during starvation.

### FAM134C degradation during starvation is controlled by mTOR in vivo

**Fig. 7.**
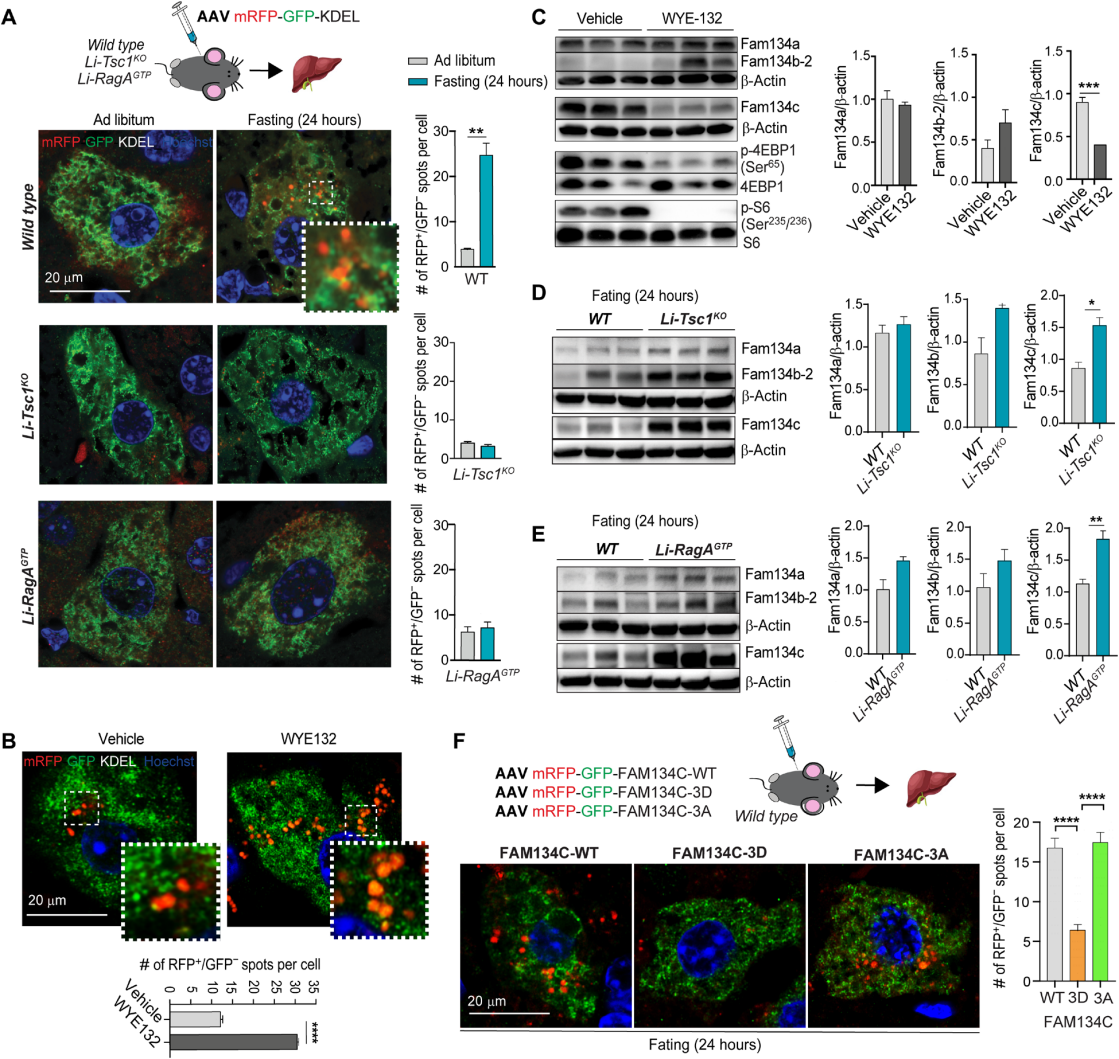
Starvation and mTOR inhibition regulate Fam134c degradation in vivo. (**A**) Representative immunofluorescence staining of RFP, GFP, and nuclei in liver sections isolated from WT, *Li-Tsc1^KO^*, and *Li-RagA^GTP^* mice injected with AAV ssRFP-GFP-KDEL kept with food at libitum or fasted for 24 hours. Scale bar, 20 μm. Quantification shows red-only positive puncta (RFP^+^) number per cell. Mean ± SEM. *N* = 3 biological replicates. *n* = 15 cells per experiment. Student’s paired *t* test: **P < 0.005. (**B**) Representative immunofluorescence staining of RFP, GFP, and nuclei in liver from WT mice injected with AAV ssRFP-GFP-KDEL and treated with WYE-132 (mTOR inhibitor) or vehicle for 24 hours. Scale bar, 20 μm. Quantification of red-only puncta (RFP^+^/GFP^−^) average per cell. Mean ± SEM. *N* = 3 biological replicates. *n* = 15 cells per experiment. Student’s paired *t* test: ****P < 0.0001. (**C**) Western blot analysis of liver extracts from WT mice treated with WYE-132 (mTOR inhibitor) or vehicle for 24 hours. β-Actin was used as a loading control. *N* = 3 biological replicates. Student’s paired *t* test: ***P < 0.0005. (**D** and **E**) Western blot analysis of liver samples isolated from WT, *Li-Tsc1^KO^* (D), or *Li-RagA^GTP^* (E) mice fasted for 24 hours. β-Actin was used as a loading control. *N* = 3 biological replicates. Student’s paired *t* test: *P < 0.05 and **P < 0.005. (**F**) Representative immunofluorescence staining of RFP, GFP, and nuclei in liver from WT mice injected with AAV mRFP-GFP-FAM134C-WT, mRFP-GFP-FAM134C-3D, or mRFP-GFP-FAM134C-3A and fasted for 24 hours. Scale bar, 20 μm. On the right, quantification shows red-only puncta (RFP^+^/GFP^−^) average per cell. Mean ± SEM. *n* = 50 cells. One-way ANOVA and Tukey’s multiple comparison test: ****P < 0.0001.

To investigate the physiological relevance of our findings, we generated adeno-associated virus (AAV) serotype 2/9 expressing the ER-phagy reporter ss-RFP-GFP-KDEL. AAV2/9-ssRFP-GFP-KDEL was systemically administered to WT C57B6 mice, and after 4 weeks, liver samples were isolated from 24-hour starved and ad libitum–fed mice. Starvation significantly increased the number of red-only puncta of infected WT hepatocytes compared with fed mice (Fig. 7A), suggesting that this tool can be used in vivo to monitor ER-phagy activation. Next, we infected mice with a liver-specific *Tsc1* KO (Li-*Tsc1*^KO^) and with a guanosine triphosphate (GTP)–bound constitutively active form of *RagA (RagA^GTP^)*; both models showed constitutive mTORC1 signaling even under fasting conditions (fig. S9, A and B) (*39*). Li-*Tsc1*^KO^ and *RagA^GTP^* hepatocytes showed a significantly reduced number of red-only puncta compared to WT, suggesting defective starvation-induced ER-phagy (Fig. 7A). WT mice infected with AAV2/9-ssRFP-GFP-KDEL treated with the mTOR inhibitor (WYE-132) for 24 hours showed a significant increase of red-only puncta compared with vehicle-treated mice (Fig. 7B). These gain- and loss-of-function data demonstrate that ER-phagy is primarily regulated by mTORC1 in liver during starvation.

Consistent with a role of FAM134C during starvation-induced ER-phagy, liver samples isolated from starved and WYE-132–treated mice showed complete degradation of Fam134c, but not of Fam134a (Fig. 7C and fig. S9C). Fam134b-2 levels were instead increased in WYE-132–treated or starved animals (Fig. 7C and fig. S9C), consistent with a transcriptional induction of *Fam134b-2* during starvation in liver (*16*). Furthermore, Fam134c, but not the other Fam134s, accumulated in liver samples isolated from starved Li-*Tsc1*^KO^ and *RagA^GTP^* mice compared to controls (Fig. 7, D and E). These data provide physiological relevance to the model by which Fam134c activity is controlled by mTORC1 levels.

To study whether Fam134c degradation is dependent on phosphorylation in vivo, we generated AAV2/9 viruses expressing WT-FAM134C, 3D-FAM134C, and 3A-FAM134C proteins fused with the RFP-GFP tandem reporter. Infected mice were analyzed after 24 hours of fasting, and we observed the appearance of numerous red-only dots in the hepatocytes infected with RFP-GFP-FAM134C-WT and RFP-GFP-FAM134C-3A but not with RFP-GFP-FAM134C-3D proteins (Fig. 7F). These data also suggest that in vivo dephosphorylation is needed for FAM134C activation.

### Deletion of FAM134C, but not of FAM134B, impairs liver lipid metabolism upon mTOR inhibition

**Fig. 8.**
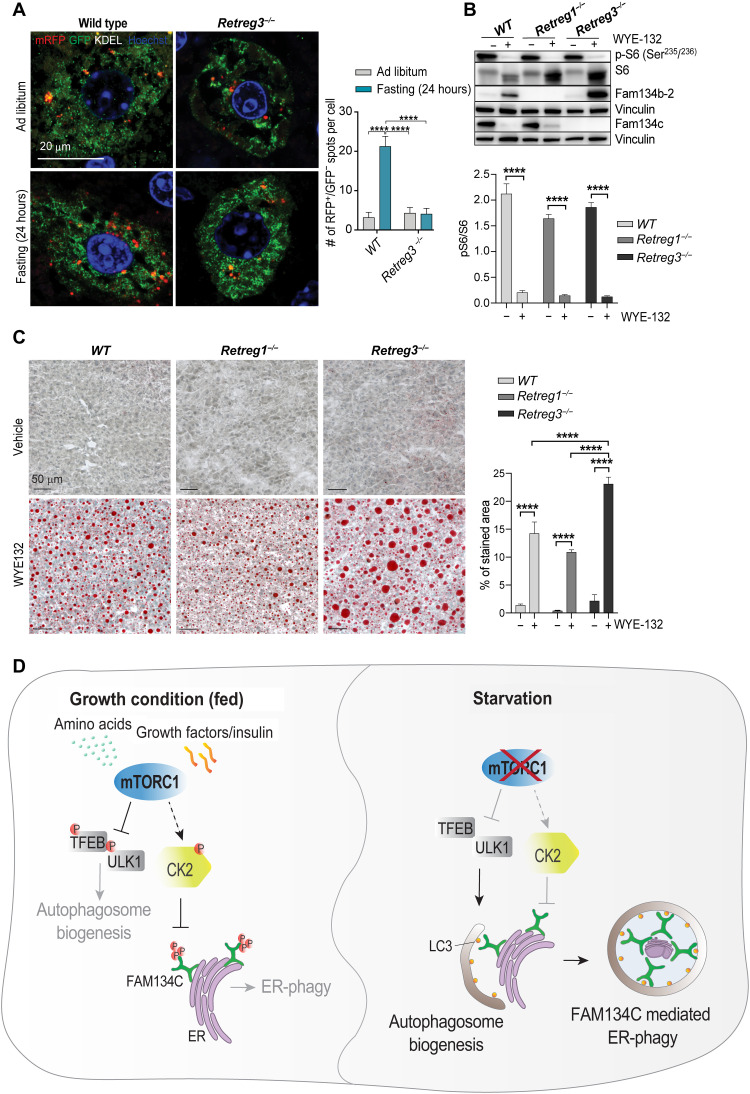
Loss of Fam134c in mouse impairs lipid metabolism in liver upon mTOR inhibition. (**A**) Representative immunofluorescence staining of RFP (red), GFP (green), and nuclei (blue) in liver from WT and *Retreg3^−/−^* mouse after ad libitum feeding or 24 hours of fasting. Scale bar, 20 μm. Quantification shows red-only positive puncta (RFP^+^) number per cell relative to ad libitum condition. Mean ± SEM. *N* = 3 biological replicates. *n* = 15 cells per experiment. Two-way ANOVA and Tukey’s multiple comparison test: ****P < 0.0001. (**B**) Representative Western blot analysis of indicated proteins in liver from WT, *Retreg1^−/−^*, and *Retreg3^−/−^* mice treated with WYE-132 (mTOR inhibitor) or vehicle for 24 hours. Vinculin was used as a loading control. On the bottom, quantification shows phosphorylated/total protein ratio. Mean ± SEM. *N* = 5 biological replicates. Two-way ANOVA and Tukey’s multiple comparison test: ****P < 0.0001. (**C**) Representative images of Oil Red O staining in WT, *Retreg1^−/−^*, and *Retreg3^−/−^* mouse liver sections. Mice were treated with WYE-132 (mTOR inhibitor) or vehicle for 24 hours. On the right, quantification of red area was reported as percentage (%) of total area. Mean ± SEM. *N* = 5 biological replicates. Two-way ANOVA and Tukey’s multiple comparison test: ****P < 0.0001. (**D**) Proposed model of FAM134C activation during starvation. The inhibition of mTORC1 concomitantly induces autophagosome biogenesis and reduction of FAM134C phosphorylation that favors autophagy of FAM134C-decorated ER fragments.

Next, we analyzed ER-phagy in mice lacking the *Retreg3* gene, which encodes Fam134c (*Retreg3^−/−^*). Control and *Retreg3^−/−^* mice were infected with AAV2/9-ss-RFP-GFP-KDEL and then kept with food ad libitum or starved for 24 hours before sacrifice. We observed robust ER-phagy induction upon starvation in control mice, but not in *Retreg3^−/−^* mice (Fig. 8A), demonstrating the importance of Fam134c during starvation-induced ER-phagy in vivo. Proteomic analysis performed in *Fam134c^KO^*, but not *Fam134b^KO^*, cells showed impaired degradation of enzymes involved in lipid biosynthesis upon Torin1 treatment (fig. S2). As mTORC1 is a critical regulator of liver lipid metabolism particularly during starvation (*3*), we analyzed lipid content in liver sections of WT, *Retreg3^−/−^*, and *Retreg1^−/−^* (encoding for Fam134b) mice treated with vehicle or mTOR inhibitor WYE-132 for 24 hours (Fig. 8, B and C). Untreated mice showed no alteration in lipid contents, independently of genotype. As expected, the pharmacological inhibition of mTOR significantly increased lipid content in the liver of WT, *Retreg3^−/−^*, and *Retreg1^−/−^* mice; however, *Retreg3^−/−^* accumulated significantly higher levels of lipids compared to WT and *Retreg1^−/−^* mice, suggesting that FAM134C-mediated ER-phagy is important for liver lipid metabolism during starvation and mTOR inhibition (Fig. 8C).

## DISCUSSION

In this work, we characterized a novel mechanism of FAM134C activation in response to starvation and mTORC1 inhibition. We propose that, during starvation, the inhibition of mTORC1 facilitates the capture of ER fragments within forming autophagosomes by enhancing the interaction between LC3/GABARAP proteins and FAM134C (Fig. 8D).

In vivo, we observed that FAM134C is quickly and very efficiently degraded in liver samples isolated from fasting and WYE-132–treated animals. Conversely, FAM134B showed an opposite response, a marked increase upon starvation and mTORC1 inhibition. One possibility is that FAM134C is involved in the initial step of ER-phagy and is regulated by a phosphorylation-dependent mechanism, whereas FAM134B accounts for a later, transcriptionally regulated, ER-phagy response to starvation. Furthermore, we demonstrated that *Fam134c* deletion in vivo affected mTOR-mediated regulation of liver lipid metabolism, a phenotype that was not evident in mice lacking *Fam134b*, demonstrating that FAM134 proteins have physiologically different roles during starvation-induced ER-phagy.

Mechanistically, we found that FAM134C binding to LC3B in response to the fed/fasting transition is regulated by CK2-mediated phosphorylation of three residues (S435, S436, and T440) located in proximity but not adjacent to the FAM134C LIR domain (F447-L450). This regulation is specific to FAM134C, because both FAM134A and FAM134B lack correspondent CK2 phosphosites; hence, their activity is not influenced by CK2. Notably, CK2 inhibition promotes ER-phagy in lung alveolar cells that express high levels of FAM134C, but not in U2OS cell line where FAM134C levels are low, suggesting that the relative expression of autophagy receptors might drive substrate specificity during autophagy.

CK2 was previously implicated in the regulation of mitophagy via phosphorylation of FUNDC1 (*40*), and also in this case, phosphorylation negatively modulates the avidity of the receptor for mATG8s. Therefore, in both cases, CK2 has the peculiar ability to negatively modulate the affinity of the autophagy receptors for mATG8s. Conversely, CK2-mediated phosphorylation of the ER-phagy receptor TEX264 at residues located in positions −1, −2, and −4 upstream the LIR domain was recently shown to enhance its binding to LC3/GABARAP proteins, hence promoting ER-phagy (*41*). The differential effects of this phosphorylation can be explained by the proximity of the phosphorylated residue to the LIR domain. The residues phosphorylated by CK2 in FAM134C interact with a region of LC3B that has a relatively neutral charge; thus, phosphorylation, by significantly increasing the local electron density, might lead to a less favorable interaction with LC3B. Conversely, the LIR domain binds an electron-poor (positively charged) LC3B region; therefore, phosphorylation of residues immediately adjacent to the LIR sequence might favor binding strength, as observed for TEX264, OPT, NIX, and BNIP3 (*41*–*44*).

CK2 is a constitutively active kinase, which is regulated at the substrate level (*33*, *45*). In yeast, CK2 substrate affinity can be influenced by mTORC1 via its effector kinase Kns1 (*35*). In mammalian cells, phosphorylation of eIF2β and Pdx1 substrates by CK2 depends on both mTORC1 and insulin signaling, respectively (*46*, *47*). In this work, we demonstrated that FAM134C is a newly identified CK2 substrate that is regulated in an mTORC1-dependent fashion.

ER-phagy deregulation has not been implicated in human diseases yet. However, we showed that impaired ER-phagy was associated with aberrant accumulation of FAM134C in liver samples isolated from mice with hyperactive mTORC1, suggesting that deregulation of FAM134C-mediated ER-phagy might contribute to the ER pathology and lipid alterations observed in diseases associated with mTORC1 hyperactivity (*48*).

Last, our work defines a mechanism of autophagy substrate selectivity that is operated during starvation-induced autophagy, providing a link between metabolic and quality control autophagy. The cross-talk between bulk and selective autophagy is still largely unexplored (*49*). The identification of new principles governing autophagy cargo selectivity holds potential implications for the treatment of many human diseases (*50*, *51*).

## MATERIALS AND METHODS

### Cell culture

U2OS TRex cells were provided by S. Blacklow (Brigham and Women’s Hospital and Harvard Medical School). The RCS cell line was a Swarm chondrosarcoma chondrocyte line. *Rptor/Rictor*-inducible KO MEFs were provided by M. N. Hall. MEF *Atg7*^KO^ cells were a gift from M. Komatsu and N. Mizushima. *Fam134c*^KO^ MEFs were provided by C. Hübner (Jena University). HEK293T cells were purchased from the American Type Culture Collection.

Cells were maintained at 37°C with 5% CO_2_ in Dulbecco’s modified Eagle’s medium (DMEM) and supplemented with 10% fetal bovine serum (FBS) and 1% penicillin and streptomycin. Complete starvation was performed in HBSS (Euroclone, ECB4006L), serum starvation was performed in DMEM (Euroclone, ECM0728), and amino acid starvation was performed in amino acid–free RPMI (US Biological, R9010-01) supplemented with 10% dialyzed FBS (Invitrogen, Thermo Fisher Scientific). Cells were reverse-transfected with Lipofectamine LTX and Plus reagent according to the manufacturer’s instructions. Plasmids are listed in table S8. Stable cell lines were generated using retroviral or lentiviral virus generated in HEK293T cells. For retrovirus, 1.3 μg of MSCV-N-Flag-HA-IRES-PURO-FAM134s and PCMVneo-mKeima-RAMP4 (table S8) vectors with the packaging plasmid viral Gag-Pol (0.95 μg) and the envelope plasmid VSV-G (0.75 μg) was transfected in a p60 dish. The medium containing lenti- or retroviral particles was spun down to eliminate dead cells. For retroviral infection, cells were spun down with retroviral suspension containing polybrene (8 μg/ml) for 90 min at 2300 rpm, followed by a medium change after 6 hours or overnight incubation. For lentivirus, 3.3 μg of plasmids pcW57-ssRFP-GFP-KDEL or pcW57-ssRFP-GFP-FAM134s (table S8) with pPAX2 packaging plasmid (2.7 μg) and pMD2.G envelope plasmid (1 μg) was cotransfected in HEK293T cells using a six-well plate. For lentivirus infection, cells were incubated with lentiviral suspension containing with polybrene (8 μg/ml; Merck, #TR-1003-G) for 24 hours. Confluent infected cell lines were then selected with the respective antibiotics.

### Generation of CRISPR clones

To deplete the genes of interest, we applied the clustered regularly interspaced short palindromic repeats (CRISPR)–CRISPR-associated protein 9 (Cas9) technology. The all-in-one vector (Sigma-Aldrich) contains specific single-guide RNA (sgRNA) under the control of a U6 promoter, a recombinant form of Cas9 protein under the control of the cytomegalovirus (CMV) promoter, and an eGFP reporter gene under the control of the SV40 promoter. RCS cells were transfected with 5 μg of all-in-one vector containing one of the following sgRNA: Atg7 sgRNA sequence: CGCTGAGGTTCACCATCCT; Fam134a sgRNA sequence: CCCCCTGAAGAACCGCACT; Fam134b sgRNA sequence: TGAGCTCTGTGGGTAAGCCAAGG; and Fam134c sgRNA sequence: CGGGCACGCTGAGCAACCG (−).

After transfection, single-cell sorting for the eGFP fluorescence was performed by FACS using BD FACSAria III. To identify the specific mutations, confluent cells were then subjected to polymerase chain reaction (PCR) analysis followed by Sanger sequencing. Selected clones were then validated by Western blotting analysis of the protein of interest.

### Chemicals

BafA1 (Euroclone, #BK54645S) was used at 100 or 200 nM for the indicated time. Torin1 (Euroclone, #BK14379S) was used at 150 nM, 250 nM, or 1 μM at different time points. CK2 inhibitor CX4945 (Selleckchem, #S2248) was used at 4 μM for 6 hours. The CK2 inhibitor SGC-CK2-1 (Tocris, #7450) was used at 50 nM for 6 hours. the mTOR inhibitor WYE-125132 (Selleckchem, #PZ0321) was used for in vivo experiments at 10 mg/kg and injected three times every 8 hours. Doxycycline (Sigma-Aldrich, #D9891) was used at 4 μg/ml and added 24 hours before the experiments. Tamoxifen (Sigma-Aldrich, #H6278) was used at 1 μM for 72 hours.

### Cloning procedures and DNA mutagenesis

To generate HA- and Myc-FAM134s plasmids, FAM134s and FAM134C mutants were cloned into pDONR223 vector (Invitrogen) using the BP Clonase Reaction Kit (Invitrogen, #11789020) and further recombined, through the LR Clonase Reaction Kit (Invitrogen, #11791020), into the GATEWAY destination vectors iTAP MSCV-N-FLAG-HA IRES-PURO or Myc-pcDNA3.1 (table S8). Myc-pcDNA3.1 GATEWAY destination vector was generated from a standard Myc-pcDNA3.1 (table S8), where the GATEWAY features (attR1-CAT/CmR-ccdB-attR2) were inset in the multiple cloning site.

For the generation of pcW57-ssRFP-GFP-FAM134s, the eGFP sequence was cleaved out from pcW57-ssRFP-GFP-KDEL (table S8) and amplified, while FAM134s sequences were amplified from Myc-FAM134s pcDNA3.1 (table S8). The In-Fusion Multiple-Insert Cloning procedure, using the In-Fusion HD Cloning Kit (Takara, # 639649), was applied to clone the two inserts into the linearized pcW57-ssRFP vector.

The C-terminal FAM134C WT and mutants were generated by amplification of the 250– to 466–amino acid sequence into the GATEWAY destination vector iTAP MSCV-N-FLAG-HA IRES-PURO or pGEX-4T1-GST (table S8). To purify C-terminal FAM134C and FAM134C mutant proteins, GST-FAM134C vectors were generated, cloning the cDNAs into the GATEWAY destination vectors pGEX-4T1 (table S8). For the generation of the retrovirus pCMVneo-mKeima-Ramp4, the Ramp4 sequence was amplified from TetOn-mCherry-eGFP-RAMP4 vector (table S8) fused at the N terminus with mKeima and inserted into pCMVneo using the In-Fusion HD Cloning Kit (Takara, # 639649) according to the manufacturer’s instructions.

To generate the AAV, the construct mRFP-GFP-KDEL or mRFP-GFP-FAM134C-WT or mRFP-GFP-FAM134C-3D was amplified from the respective vectors (table S8). The pTigem plasmid (table S8) containing the inverted terminal repeats of AAV2, the kanamycin resistance sequences, and the CMV promoter was linearized, and inserts were cloned using the In-Fusion HD Cloning Kit.

For FAM134C mutagenesis, primers were designed using the QuikChange Primer Design tool (Agilent). cDNA mutations were generated via PCR site-directed mutagenesis using QuikChange II mutagenesis kit (Agilent), according to the manufacturer’s protocol. Transformation was performed in DH5α Competent Cells (Invitrogen).

### Immunoblotting of total cell lysates

Cells were washed twice with phosphate-buffered saline (PBS) and then scraped in radioimmunoprecipitation assay (RIPA) lysis buffer [20 mM tris (pH 8.0), 150 mM NaCl, 0.1% SDS, 1% NP-40, and 0.5% sodium deoxycholate] supplemented with PhosSTOP and EDTA-free protease inhibitor tablets with 1× final concentration (Roche). Cell lysates were incubated on ice for 20 min; then, the soluble fraction was isolated by centrifugation at 15,000 rpm for 20 min at 4°C. Protein concentration was measured using the colorimetric bicinchoninic acid (BCA) protein assay kit (Pierce Chemical Co.). Protein extracts (25 to 50 μg) were separated by SDS–polyacrylamide gel electrophoresis (SDS-PAGE) and transferred onto polyvinylidene difluoride (Merck Millipore) or nitrocellulose membrane (PerkinElmer). Primary antibodies used are listed in table S9. Horseradish peroxidase–conjugated goat anti-mouse or anti-rabbit immunoglobulin G antibody was used at 1:2000 (Vector Laboratories). Signal detection was performed using ECL Star Enhanced Chemiluminescent Substrate (Euroclone) according to the manufacturer’s protocol. The Western blotting images were acquired using the ChemiDoc-lt imaging system (UVP).

### Coimmunoprecipitation

HEK293T cells were cotransfected with pMSCVneo-GFP-LC3 plasmid together with 3D-Myc-FAM134C or 3A-Myc-FAM134C. After 24 hours of transfection, cells were incubated for 8 hours with BafA1 (100 nM), harvested, and lysed in RIPA buffer containing protease and phosphatase inhibitor cocktails. Lysates were centrifuged at 14,800 rpm for 20 min and quantified, and 500 μg was incubated overnight at 4°C with 10 μl of anti-Myc agarose bead slurry (Diagnostic Brokers Associated, yta-100). Beads were then washed three times in RIPA buffer, resuspended in Laemmli buffer, and boiled. The 50% of the immunoprecipitated fraction was loaded on SDS-PAGE, and Western blotting analysis was performed using the anti-GFP and anti-Myc antibodies.

Stable cell lines for FAM134s were induced with doxycycline (1 μg/ml) for 24 hours. U2OS cells expressing FAM134s were harvested in 50 mM tris-HCl (pH 8.0), 120 mM NaCl, 1% (v/v) NP-40, and complete protease inhibitor cocktail in a confluent 15-cm-diameter petri dish. Lysates were cleared by centrifugation at 10,000*g* for 10 min and incubated overnight at 4°C with monoclonal anti–HA-agarose antibody (Thermo Fisher Scientific, #26181), previously conjugated to Protein G Sepharose beads (Thermo Fisher Scientific, #15918014). Beads were then washed three times in 0.1% (v/v) NP-40. Proteins were eluted by boiling in Laemmli buffer and loaded on SDS-PAGE for Western blot analysis or processed for MS analysis.

### Immunofluorescence

Cells were fixed for 15 min in 4% paraformaldehyde (PFA) in PBS and permeabilized for 20 min in blocking buffer [0.05% (w/v) saponin, 0.5% (w/v) bovine serum albumin (BSA), 50 mM NH_4_Cl, and 0.02% NaN_3_ in PBS]. Cells were incubated overnight in a humid chamber with primary antibodies (table S9), washed three times in PBS, and incubated for 1 hour at room temperature with the secondary (Alexa Fluor–labeled) antibodies. After three washes in PBS, cells were incubated for 20 min with Hoechst 33342 (1 μg/ml) and finally mounted in Mowiol. Anti-mouse or anti-rabbit Alexa Fluor–labeled antibody (1:1000; Thermo Fisher Scientific) was used. All confocal experiments were acquired with an LSM 880 confocal microscope (Carl Zeiss) equipped with a 63× or 100× objective and using a slice thickness of 0.5 μm. All the quantifications were performed using ImageJ plugins.

### ER-phagy assays: ssRFP-GFP-KDEL and mKeima-RAMP4

*Fam134c^KO^* MEF and *Fam134^KOs^* RCS cell lines were infected with lentivirus pCW57-CMV-ssRFP-GFP-KDEL to generate ER-phagy reporter-inducible cell lines. The expression of the plasmid was induced with doxycycline (4 μg/ml; 24 hours), and starvations or Torin1 treatment was applied at different time points, as indicated. Cells were either collected in PBS and the fluorescence was analyzed with BD FACSAria III or fixed in 4% PFA for 15 min to quantify the number of red-only puncta after image acquisition. For FACS analysis, 10,000 fluorescent events (both red and green) were collected and red fluorescent shift was analyzed by applying a specific threshold based on the untreated samples.

### Protein expression and purification from bacteria

FAM134C (250 to 466) was cloned into pGEX6P1 and expressed as glutathione *S*-transferase (GST) fusion proteins in *Escherichia coli* BL21 DE3. Cells were lysed by sonication in lysis buffer [20 mM tris-HCl (pH 7.5), 10 mM EDTA (pH 8), 5 mM EGTA, 150 nM NaCl, and 1% Triton X-100], and the lysate was cleared by centrifugation. The expressed protein was purified by 20 nM glutathione (GSH) in 10 mM tris (pH 8), 1 mM dithiothreitol, 1 mM EDTA, and 300 nM NaCl with final pH 8.

### In vitro phosphorylation assays

To perform the FAM134C phosphorylation assay, different amounts of WT or mutant recombinant FAM134C C-terminal proteins were incubated with recombinant tetrameric CK2 (7 ng) for 20 min at 30°C in a total volume of 20 μl of radioactive phosphorylation buffer containing 50 mM tris-HCl (pH 7.5), 10 mM MgCl_2_, 0.1 M NaCl, and 10 μM [γ-^33^P]adenosine triphosphate (ATP) (Hartmann Analytic) at 5000 counts per minute (cpm)/pmol. Reactions were stopped by adding Laemmli buffer. Proteins were resolved onto 11% SDS-PAGE, stained with Coomassie blue, and analyzed by digital autoradiography (CyclonePlus Storage Phosphor System, PerkinElmer) to detect and quantify radioactive bands.

For CK2 kinase assay, CK2 activity was measured toward the model peptide substrate RRRADDSDDDDD (0.1 mM), as elsewhere described (*52*). For the mTOR kinase assays, the RRR-QAASNFKSPVKTIR peptide corresponding to CK2β human sequence [203–215] and the control peptide RRR-NQVFLGFTYVAP, corresponding to p70 S6 kinase human sequence [382–393], were synthetized using a multiple peptides synthesizer (SyroII, MultiSynTech GmbH) (the three additional arginine residues at the N terminus allow peptide binding to phosphocellulose paper upon kinase assays). Recombinant truncated mTOR (1362–end, Millipore) was incubated at 30°C in a total volume of 25 μl of a radioactive phosphorylation mixture containing 50 mM Hepes (pH 7.5), 1 mM EGTA, 3 mM MnCl_2_, and 100 μM [γ-^33^P]ATP (Hartmann Analytic) at 3000 cpm/pmol. Reactions were stopped by sample absorption on phosphocellulose papers. Papers were washed three times with 75 mM phosphoric acid and counted in a scintillation counter (PerkinElmer).

### MS proteomics and phosphoproteomics

For the proteomic experiment, cells were plated into six-well plates and incubated for 1 hour in complete DMEM the following day. Afterward, cells were washed twice with PBS and then incubated for 20 min in RIPA lysis buffer [20 mM tris (pH 8.0), 150 mM NaCl, 0.1% SDS, 1% NP-40, and 0.5% sodium deoxycholate] supplemented with PhosSTOP and EDTA-free protease inhibitor tablets. Cell lysates were then centrifuged at 15,000 rpm for 20 min at 4°C. The soluble fraction was collected, and protein concentration was measured using the colorimetric BCA protein assay kit (Pierce Chemical Co., Boston, MA, USA). Protein extracts (50 μg) were then processed for MS analysis.

For phosphorylation analysis, HA-FAM134C U2OS stable cell lines were induced with doxycycline (4 μg/ml) for 24 hours. Cells were starved for 5 hours (HBSS containing 200 nM BafA1) and stimulated for 20 min with complete DMEM supplemented with 1× essential (Invitrogen, #11130051) and nonessential amino acids (Invitrogen, #11140050), 1× glutamine (Invitrogen, #25030081), and 100 nM insulin (Merck, #I9278). Cells were washed twice with PBS, scraped, and incubated for 20 min with RIPA lysis buffer supplemented with PhosSTOP and EDTA-free protease inhibitor tablets.

All the experiments were performed in a labeling-free setting. For full proteomes in FAM134-overexpressing cells and IP-MS interactomes, proteins were precipitated in acetone and then reduced and alkylated in a solution of 6 M guanidine-HCl, 5 mM tris(2-carboxyethyl)phosphine (TCEP), and 20 mM chloroacetamide. Peptides were obtained by digesting proteins with LysC (Wako) for 3 hours at 37°C and with the endopeptidase sequencing-grade trypsin (Promega) overnight at 37°C. Collected peptide mixtures were concentrated and desalted using the Stop and Go Extraction (STAGE) technique (*53*). For full proteomes in RCS cells, peptides were obtained using the iST Kit from PreOmics. For phosphoproteomes, U2OS cells were lysed [2% SDS, 50 mM tris-HCl (pH 8), 150 mM NaCl, 10 mM TCEP, and 40 mM chloracetamide], heated at 95°C for 10 min, and sonicated with Sonics Vibra-Cell (1-s ON/1-s OFF pulse for 30 s using 30% amplitude). The equivalent of 1 mg of protein lysate was precipitated by methanol/chloroform using four volumes of ice-cold methanol, one volume of chloroform, and three volumes of water. The mixture was centrifuged at 20,000*g* for 30 min, the upper aqueous phase was removed, and three volumes of ice-cold methanol were added. Proteins were pelleted by centrifugation and washed twice with one volume of ice-cold methanol and air-dried. The resulting protein pellet was resuspended in 8 M urea with 10 mM 4-(2-hydroxyethyl)-1-piperazinepropanesulfonic acid (EPPS) (pH 8.2). For digestion, 300 μg of proteins was diluted to 1 M urea and incubated 1:50 (w/w) ratio with LysC (Wako Chemicals) for 3 hours and 1:100 (w/w) ratio with sequencing-grade trypsin (Promega) overnight. The reaction was acidified using trifluoroacetic acid (TFA) (0.5%) and purified using Sep-Pak tC18 (Waters, 50 mg) according to the manufacturer’s protocol. Phosphopeptides were enriched using Titansphere Phos-TiO beads (GL Sciences Inc.) according to the manufacturer’s protocol. Phosphopeptides were cleaned up by C8 stage tip and fractionated using C18 stage tips. After washing (80% acetonitrile) and after an equilibration step with 0.1% TFA, peptides were loaded on C18 stage tips in 0.1% TFA solution and washed twice with 0.1% TFA in water. Samples were vacuum-dried for liquid chromatography (LC)–MS measurements.

Instruments for LC-MS/MS analysis consisted of NanoLC 1200 coupled via a nanoelectrospray ionization source to the quadrupole-based Q Exactive HF benchtop mass spectrometer. Peptide separation was carried out according to their hydrophobicity on a homemade chromatographic column, 75-μm inside diameter, 8-μm tip, bed-packed with Reprosil-PUR (C18-AQ), 1.9-μm particle size, 120-Å pore size, using a binary buffer system consisting of solution A (0.1% formic acid) and solution B (80% acetonitrile and 0.1% formic acid). Runs of 120 min after loading were used for proteome samples, with a constant flow rate of 300 nl/min. After sample loading, run started at 5% buffer B for 5 min, followed by a series of linear gradients, from 5 to 30% B in 90 min, then a 10-min step to reach 50% and a 5-min step to reach 95%. This last step was maintained for 10 min. Q Exactive HF settings are as follows: MS spectra were acquired using 3E6 as an AGC target, a maximal injection time of 20 ms, and a 120,000 resolution at 200 mass/charge ratio (*m/z*). The mass spectrometer was operated in a data-dependent Top20 mode with subsequent acquisition of higher-energy collisional dissociation fragmentation MS/MS spectra of the top 20 most intense peaks. Resolution, for MS/MS spectra, was set to 15,000 at 200 *m*/*z*, AGC target to 1E5, maximum injection time to 20 ms, and the isolation window to 1.6 Th. The intensity threshold was set at 2.0 E4 and dynamic exclusion at 30 s.

### Data analysis

All experiments were performed in at least three independent biological replicates. For MS, all acquired raw files were processed using MaxQuant (1.6.2.10) and using the implemented Andromeda search engine. For protein assignment, spectra were correlated with the UniProt Homo Sapiens and Rattus Norvegicus database (v. 2019) including a list of common contaminants. Searches were performed with tryptic specifications and default settings for mass tolerances for MS and MS/MS spectra. Carbamidomethyl at cysteine residues was set as a fixed modification, while oxidations at methionine, acetylation at the N terminus, and phosphorylation STY (in the case of phosphoproteomes) were defined as variable modifications. The minimal peptide length was set to seven amino acids, and the false discovery rate (FDR) for proteins and peptide spectrum matches to 1%. The match-between-run feature with a time window of 0.7 min was used. For further analysis, the Perseus software (1.6.2.3) was used and first filtered for contaminants and reverse entries as well as proteins that were only identified by a modified peptide. For full proteomes and IP interactomes, the label-free quantification ratios were logarithmized, grouped, and filtered for minimum valid number (minimum of three in at least one group). Missing values have been replaced by random numbers that are drawn from a normal distribution.

Data obtained for the total proteome analysis in U2OS and RCS cells are reported in tables S1 and S2, respectively. For ER proteome analysis, ER proteins were downloaded from the Subcellular Location section of The Human Protein Atlas database (www.proteinatlas.org/about/download). Data were filtered to keep only ER and ribosomal proteins (table S3). In Fig. 1H (proteome analysis in RCS), the proteins found to be significantly modulated by Torin1 treatment in control cells were used for downstream analyses in cells with the different genotypes (table S5). For phosphoproteomes, intensity values were filtered for the sites that have localization probability of >0.75. After this, the values were logarithmized, grouped, and filtered for minimum valid number (minimum of three in at least one group). Last, the intensities were normalized by subtracting the median intensity of each sample. Significantly regulated proteins between conditions were determined by Student’s *t* test using FDR < 0.05 as threshold. Data are available via ProteomeXchange with identifier PXD030451 (username: reviewer_pxd030451@ebi.ac.uk; password: KoKm7mWD).

### MD simulations

The three-dimensional (3D) structure of LC3B (PDB ID 6LAN) was obtained from the Research Collaboratory for Structural Bioinformatics Protein Data Bank (RCSB PDB) (*54*). The side chains of residues D48, E62, and S90 were not univocally resolved, allowing us to choose by visual inspection the side chain conformations B, A, and A for these three residues, respectively. The first nine residues, belonging to CCDC50, were removed to obtain the LC3 WT. The 3D structure of FAM134C_P434-P460 was built by means of the MODELLER suite using the FAM134B structure with PDB ID 7BRQ as template. The starting 3D structure of the complex formed by FAM134C_P434-P460 and LC3B was obtained by using the molecular docking program HADDOCK 2.4 (*55*). In the docking calculation, we defined the active residues a priori, i.e., F447 and L450 for FAM134C_P434-P460 and I23, F52, and I66 for LC3B. To select the best binding pose, we measured the distance between FAM134C’s L438 and LC3b’s L71, a hydrophobic interaction predicted to be crucial for the binding mechanism. The binding complex obtained from docking was then solvated using a box of TIP3P water model and a salinity of 0.15 M NaCl. The N terminus and C terminus of FAM134C_P434-P460 were capped with an acetyl and a methyl amino-protecting group, respectively. The Amber ff99sb-ildn force field was used (*56*) for the MD calculations that were run using the GROMACS 2020.5 engine. Each simulation box underwent a thermalization cycle with smoothly decreasing restraints on heavy atoms to gently equilibrate the structure. In detail, we used the following protocol: Heating the system from 100 to 300 K by increasing the temperature by 50 K each step composed of 1 ns of canonical ensemble (NVT) simulation followed by 1 ns of isothermal–isobaric (NPT) ensemble simulation. During the thermalization, the V-rescale thermostat was used, whereas the Langevin dynamics temperature control scheme was used in the production runs. Periodic boundary conditions were applied, and the particle mesh Ewald method was used to treat long-range electrostatic interactions (*57*). For short-range interactions, a cutoff distance of 1.0 nm was applied. The pressure was fixed at a reference value equal to 1 bar by means of the Parrinello-Rahman barostat (*58*).

### Parallel-tempering MD

We used 70 independent replicas of 500 ns of the FAM134C_P434-P460/LC3b system. Each replica was simulated at a different temperature to cover the temperature range from 300 to 450 K. The temperature of each replica was defined using the formula reported by Prakash *et al.* (*59*). Positional restraints were applied on the secondary structure Cαs of LC3B and on D445-P460’s Cαs of FAM134C_P434-P460. For the production phase, we used the same protocol previously reported for the MD simulations.

### FAM134C_P434-P460/LC3b binding interface evaluation

The protein-protein interactions established by the residues at the FAM134C_P434-P460/LC3B’s interface were assessed by calculating their frequency of occurrence using the PLOT NA routine of Drug Discovery Tool (*60*) and displayed as histograms. We set a distance cutoff value of 4 Å to define two interacting residues.

### Animal housing

All mice in this study were housed under specific pathogen–free conditions, at 22°C, and with 12-hour dark/12-hour light cycles (light cycle from 8:00 a.m. to 8:00 p.m.). Mice were fed with a standard chow diet and were of pure C57BL/6 background. All mice were observed weekly by trained personnel. All studies on mice were conducted in strict accordance with the Institutional Guidelines for animal research and approved by the Italian Ministry of Health, Department of Public Health, Animal Health, Nutrition, and Food Safety in accordance to the law on animal experimentation (D.Lgs. 26/2014). Furthermore, all animal treatments were reviewed and approved in advance by the Ethics Committee at the Telethon Institute of Genetics and Medicine (TIGEM) Institute (Pozzuoli, Italy) or according to protocols approved by the Centro Nacional de Investigaciones Oncológicas (CNIO)-Instituto de Salud Carlos III Ethics Committee for Research and Animal Welfare (CEIyBA) at the CNIO (Madrid, Spain).

All mice used were maintained in a C57BL/6 strain background. To minimize variability, mice belonging to the same litter were grouped on the basis of their genotype.

### Animal procedures

For fasting experiments, mice were separated in clean cages without access to food from 8 a.m. to 8 a.m. the following day. For the experiments with the mTOR inhibitor WYE-125132, mice were starved from 6 p.m. to 1 p.m. the following day and then injected intraperitoneally with WYE-125132 or vehicle (10 mg/kg) [30% PEG400 (polyethylene glycol, molecular weight 400), 0.5% Tween 80, and 5% propylene glycol] and refed with chow 1 hour after the injections. Vehicle or WYE-125132 was injected three times, every 8 hours, for a total treatment of 24 hours.

For Western blotting, 25 mg of liver was homogenized in 1 ml of RIPA buffer containing protease and phosphatase inhibitor cocktails using a Tissue Lyser II homogenizer (QIAGEN) and left on a rotating wheel overnight at 4°C. Then, pellets were discarded by spinning down for 30 min at maximum speed, and BCA assay was performed on supernatants to determine protein concentration.

Immunostaining of RFP and GFP was performed on 10-μm-thick formalin-fixed and paraffin-embedded liver sections. Sections were permeabilized in 3% (w/v) BSA, 0.3% Triton X-100 in PBS for 20 min and then incubated with 10% goat serum in PBS for 1 hour. Primary antibodies were incubated overnight at 4°C. Sections were washed three times with 0.1% BSA in PBS and then incubated for 1 hour with secondary antibodies Alexa Fluor–conjugated and 4′,6-diamidino-2-phenylindole (DAPI). Sections were mounted in Mowiol mounting medium and analyzed using a superresolution confocal LSM 880 microscope (Carl Zeiss).

For Oil Red O staining, tissues were embedded in O.C.T. (optimal cutting temperature) compound and cut into 10-μm sections, fixed in formalin, and stained following the IHC World protocol. To quantify the Oil Red O–positive area, images were processed through the threshold tool of the ImageJ software to segment out and measure the staining areas as percentage of total area.

### Generation of *Retreg3* KO mice

Mice carrying *Retreg3* KO were generated using the European Conditional Mouse Mutagenesis/KnockOut Mouse Project (EUCOMM/KOMP)-CSD “Knockout-First” ES cell resource at MRC Harwell Institute. The targeting cassette of the transgenic *Retreg3* allele contains a promotorless β-galactosidase and neomycin resistance genes flanked by [LoxP-flippase (FLP) recognition target] and LoxP sites flanking exon 2 of the *Retreg3* gene. Excision of exon 2 was achieved by crossing the transgenic mice carrying the transgenic cassette with germline Cre-expressing mice.

Genotyping strategy consists of a three-primer PCR setup by designing a mutant reverse primer (5mut-R1, GAACTTCGGAATAGGAACTTCG) that sits in the 5′ FRT site and a forward primer (Fam134c-5arm-WTF, GGCATTAATATACACAATAGCACAA) to give a mutant-specific band [119 base pairs (bp)] when the cassette is present, or a WT band (269 bp) when the cassette is not present and a reverse primer (Fam134c-Crit-WTR, GGCACGTGGATTTCTGAGTT) sitting in exon 2 is used. When the cassette is present, the product between Fam134c-5arm-WTF and Fam134c-Crit-WTR is too large to be amplified under standard PCR conditions.

Mice with constitutive nutrient and growth factor signaling in liver, *Li-TSC1^KO^* and *Li-RagA^GTP^*, were achieved by breeding *TSC1^flox/flox^* mice and *RagA^GTP/flox^* mice with Albumin-Cre transgenic mice, as described in (*39*).

### AAV vector production and injection

AAV2/9 vectors were produced by InnovaVector (www.innovavector.eu/) by triple transfection of HEK293 cells with (i) mRFP-GFP-KDEL, mRFP-GFP-Fam134c-WT, mRFP-GFP-Fam134c-3D, or mRFP-GFP-Fam134c-3A; (ii) pTigem Helper, which supplies the adenoviral genes for replication; and (iii) pTigem Rep/Cap, which contains the gene sequence for the replication protein (rep) of AAV2 and for the capsid (cap) proteins of the selected serotype. AAV2/9 vectors were then purified by two rounds of CsCl_2_ gradients. Physical titers of the viral preparations (genome copies per milliliter) were determined by real-time PCR quantification using TaqMan (Applied Biosystems) and dot-blot analysis. The final titer of each preparation was calculated as the average between the PCR quantification and dot-blot results.

For the AAV injections, following anesthesia with isoflurane, 1 × 10^12^ viral particles in 100 μl of PBS were injected into the retro-orbital venous plexus of 8-week-old mice with a 27-gauge syringe. Mice were euthanized 4 weeks after the injection.

### Statistical analysis

All statistical data were calculated using GraphPad Prism 7 except for proteomic and phosphoproteomic data that were calculated by Perseus. Data comparisons were performed using Student’s *t* test or analysis of variance (ANOVA) (one-way or two-way) with Sidak’s and/or Tukey’s multiple comparison test. *P* values of <0.05 were considered significant. All experiments were repeated at least three times. The exact sample size for each experiment is reported as *N* in the caption of each figure. Results are presented as the mean ± SEM.

## Notes

View/request a protocol for this paper from *Bio-protocol*.
