# Supplementary material for: Phosphorylation of FAM134C by CK2 controls starvation-induced ER-phagy
Source: Sci Adv. 2022 Aug 31;8(35):eabo1215. doi: 10.1126/sciadv.abo1215 (PMC9432840; doi:10.1126/sciadv.abo1215)
Supplement: Supplementary file 1 — Figs. S1 to S9 [file sciadv.abo1215_sm.pdf]

Science Advances
AAAS

Supplementary Materials for

Phosphorylation of FAM134C by CK2 controls starvation-induced ER-phagy

Giorgia Di Lorenzo *et al.*

Corresponding author: Paolo Grumati, p.grumati@tigem.it; Carmine Settembre, settembre@tigem.it

Sci. Adv. **8**, eabo1215 (2022)
DOI: 10.1126/sciadv.abo1215

The PDF file includes:

Figs. S1 to S9
Legends for tables S1 to S9

Other Supplementary Materials for this manuscript includes the following:

Tables S1 to S9

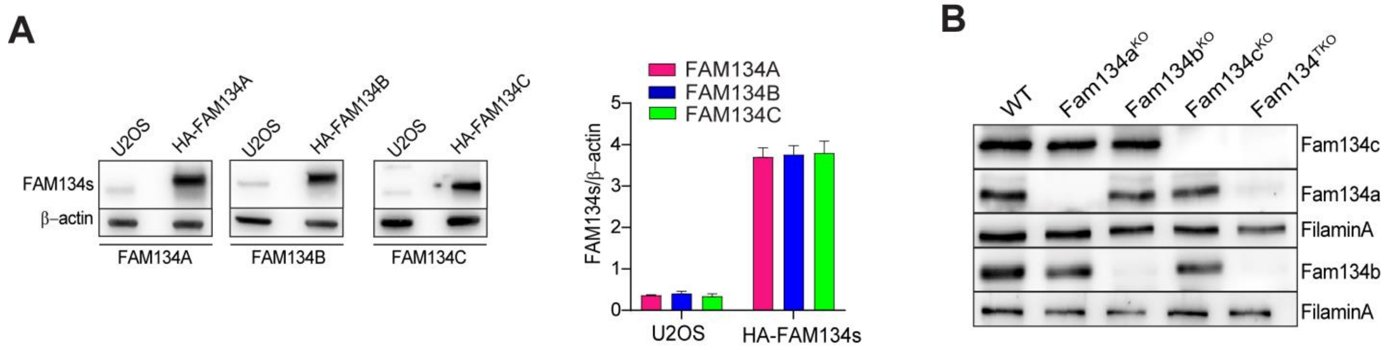

Fig. S1. FAM134s overexpressing and KO cell lines. (A) Representative western blot analysis of FAM134s in U2OS WT and overexpressing doxycycline inducible HA-FAM134-A, -B, or -C. ß-actin was used as a loading control. On the right, quantification showing HA-FAM134s/ß-actin ratio. Mean ± SEM. N=3 biological replicates. **B)** Representative western blot analysis of FAM134s in RCS WT and *Fam134-a*, -*b*, -*c* ^{KO} and -^{TKO}. Filamin A was used as a loading control.

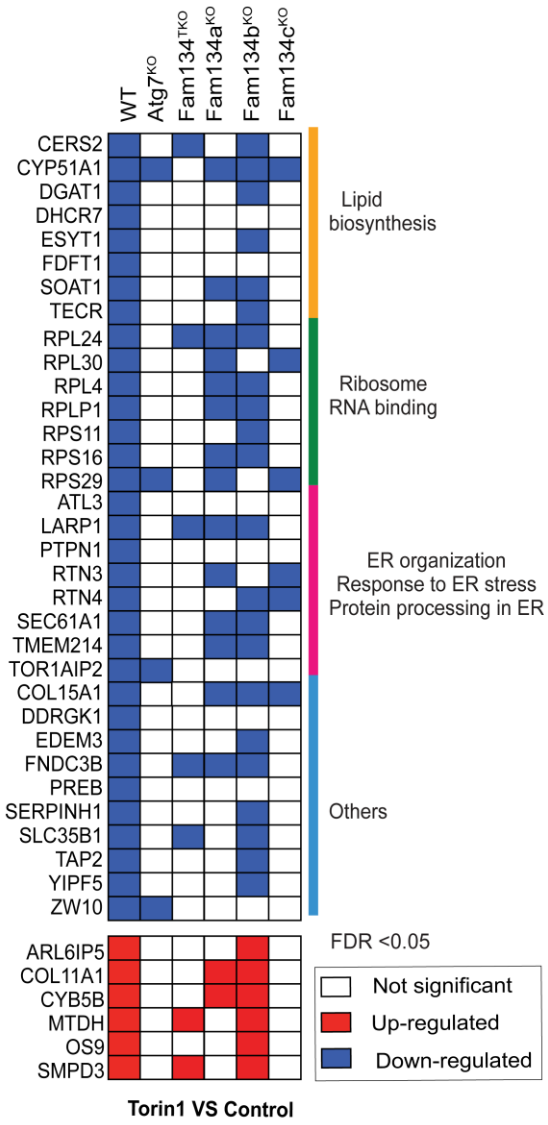

Fig. S2. Contribution of Fam134 proteins in the regulation of ER-proteome upon mTOR inhibition. Proteomic analysis performed in RCS cells with the indicated genotypes. The table shows significantly down- (blue), up- (red) and not significantly (white) regulated ER-resident proteins (selected using the ER-protein-atlas extended database) upon Torin1 treatment (12h; 150nM).

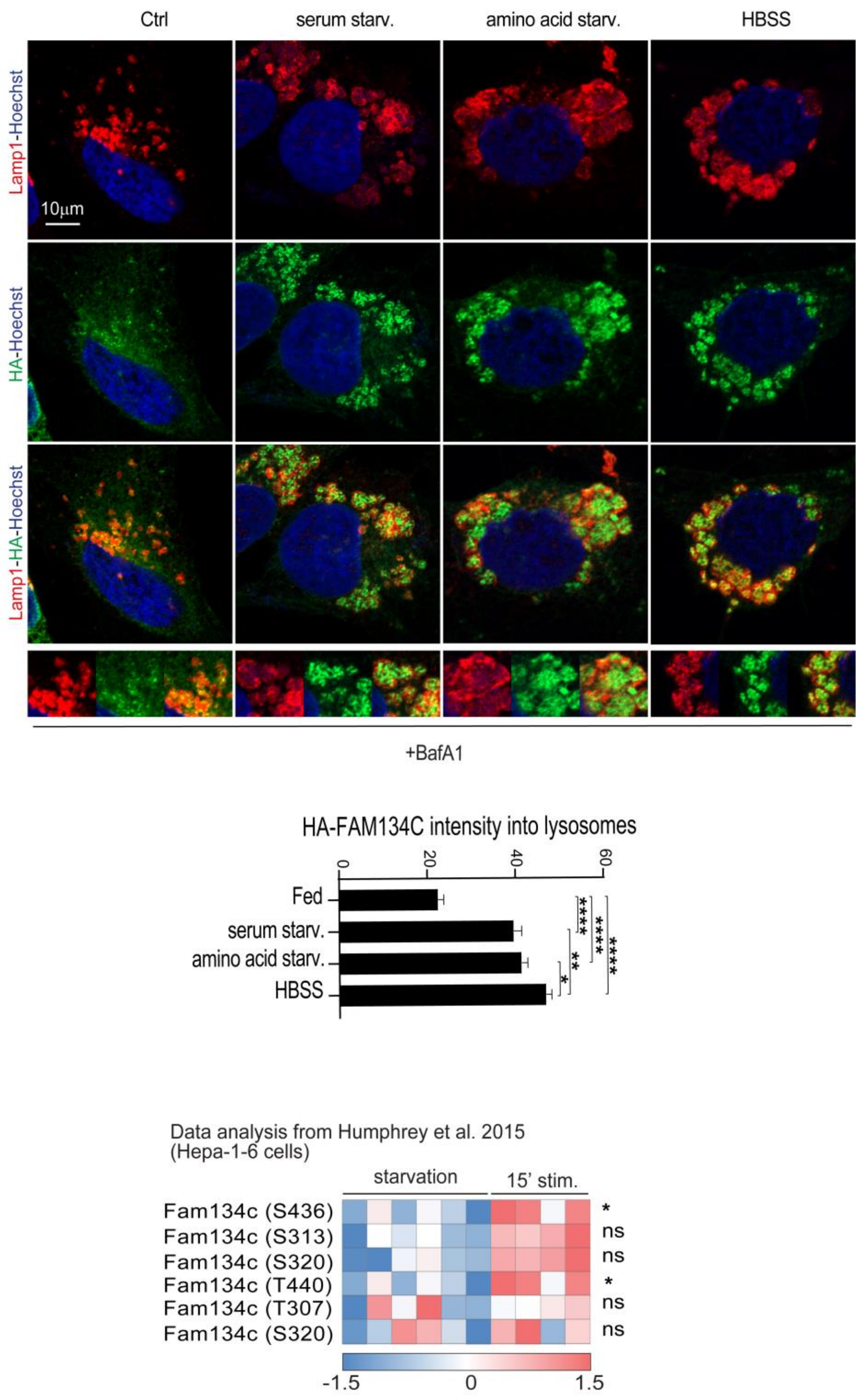

Fig. S3. mTOR regulates FAM134C lysosomal delivery through phosphorylation. (A) Representative images of immunofluorescence staining for HA (green), LAMP1 (red) and nuclei (blue) in U2OS overexpressing doxycycline inducible HA-FAM134C cultured in growth medium (Ctrl) or starved for 8h in the presence of Bafilomycin (BafA1; 100nM). Scale bar 10μm. On the bottom, quantification shows HA-FAM134C fluorescence intensity into LAMP1 decorated lysosomes. Mean ± SEM. *N*=3 biological replicates. *n*=15 cells/experiment. One-way Anova, Tukey's multiple comparison test, *P < 0.05, **P<0.005, ****P < 0.0001. **(B)** Data analysis from (*28*). Heat-map of the phosphorylation levels of FAM134C residues in serum deprived *vs* Insulin

stimulated (100nM; 15 min) Hepa-1-6 cells. Heat-map is colour coded according to the intensity level of the phosphorylated site. *N*=6 (starvation) and *N*=4 (stimulation) biological replicates. FDR: *P < 0.05, ns= not significant.

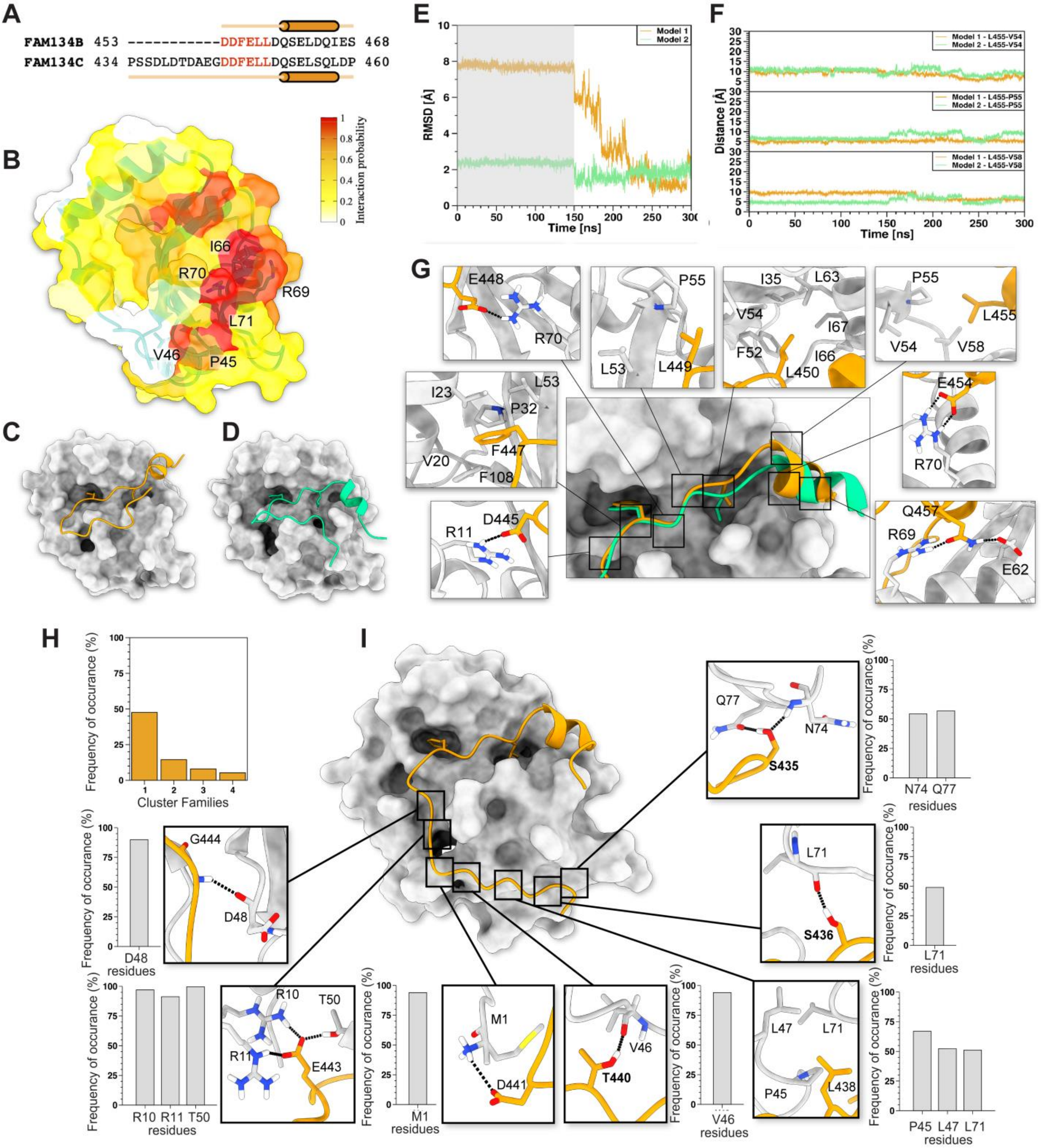

J

	HADDOCK score
3D	-157.1
S435D	-156.0
S436D	-156.0
T440D	-172.2
3A	-177.2
WT	-183.3

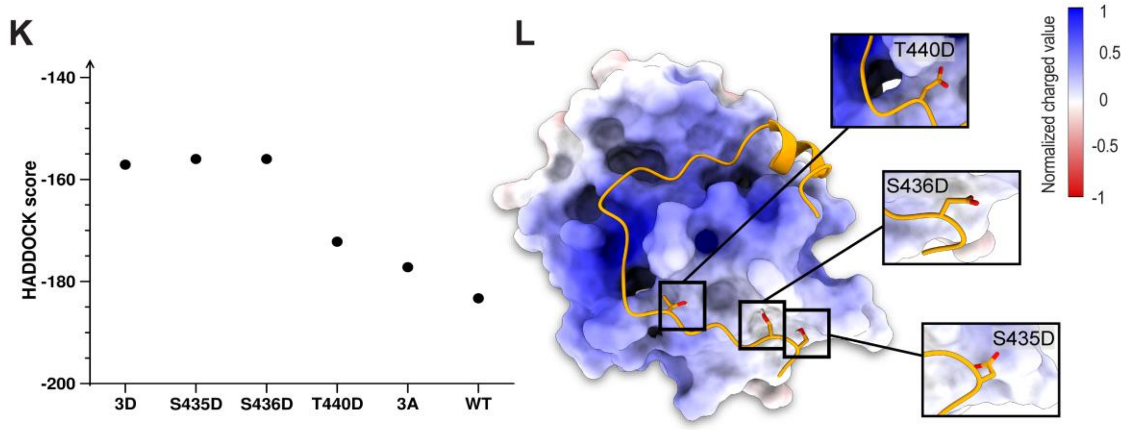

Fig. S4. Structural analysis of FAM134C and LC3B. **(A)** Sequence alignment of FAM134B and FAM134C (FAM134C_P434-P460), limited to the region including the LIR (shown in red). The secondary structure element is represented in orange. **(B)** Interaction probability of FAM134C's L438 with LC3B's residues, projected onto the solvent accessible surface of LC3B. The interaction probability for each amino acid residue of LC3B has been highlighted through the color scheme on the right side. **(C)** Representation of the first FAM134C_P434-P460/LC3B model. FAM134C_P434-P460 is colored in orange, LC3B is in grey and represented as a solvent accessible surface. **(D)** Representation of the second FAM134C_P434-P460/LC3B model. FAM134C_P434-P460 is colored in green, while LC3B is in grey and represented as a solvent accessible surface. **(E)** Plot of the RMSD computed as a function of simulation time for the Cα atoms of FAM134C_P434-P460's D445-P460. The first 150 ns represents the equilibration phase and are shown in grey. **(F)** Plot reporting the L455-V54, L455-P55, and L455-V58 distances as a function of the simulation time. **(G)** Representation of the most populated FAM134C_P434-P460/LC3B binding mode obtained from MD calculations on the two models. The insets depict the most relevant interactions established between FAM134C and LC3B. The two binding models **(C)** and **(D)** are very similar, therefore for the sake of clarity only the first one **(C)** is displayed. FAM134C's residues are displayed in orange, while LC3B is in grey and shown in the central panel as a solvent accessible surface. **(H)** Histogram reporting the results of the conformational cluster analysis performed on the 300 K trajectory associated with the FAM134C_P434-P460/LC3B PT-MD simulations. **(I)** Representation of the FAM134C_P434-P460/LC3B complex structure representing the most populated cluster family obtained from the PT-MD simulations. The insets depict the most relevant interactions established between FAM134C's P434-G444 and LC3B along with their frequency of occurrence calculated from the PT-MD simulations. FAM134C is in orange, while LC3B is shown through its solvent accessible surface and colored in grey. **(J)** The affinity scores of the best binding modes identified as those with the lowest RMSD with respect to the FAM134C_P434-P460/LC3B complex reported in Fig. 2C. **(K)** Plot of the WT and mutant forms of FAM134 as a function of the docking score. **(L)** Structural analysis of the 3D FAM134C_P434-P460/LC3B complex. Three insets display the position of S435D, S436D, and T440D mutations on the surface of LC3B. S435D, S436D, and T440D were represented in ribbon and colored in orange. LC3B was represented through its solvent accessible surface, colored according to the local electrostatic potential map values following the color gradient reported on Fig. 2B.

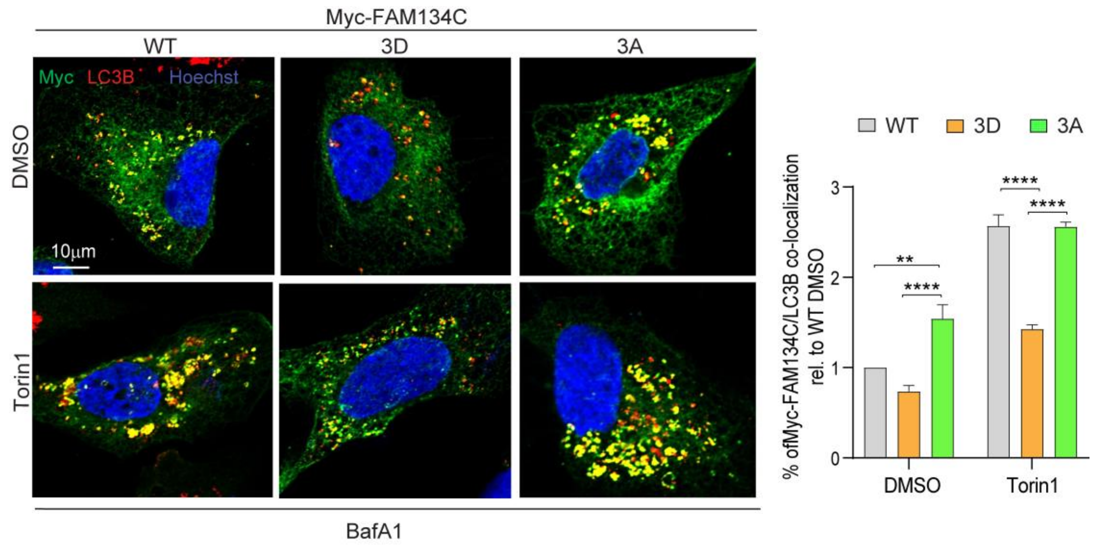

Fig. S5. Phosphorylation regulate FAM134C-LC3 co-localization. Representative immunofluorescence staining of Myc (green), LC3B (red) and nuclei (blue) in U2OS cells transfected with Myc-FAM134C wild-type (WT), phospho-mimetic (3D) and phospho-mutant (3A). Cells were treated for 2h with Bafilomycin A1 (BafA1; 100nM) and then DMSO or Torin1 (250nM) was added for 6h. Scale bar 10µm. On the right, quantification shows percentage (%) of co-localization of Myc-FAM134 with LC3B relative to WT (DMSO). Bar graph shows mean ± SEM. N=3 biological replicates. n=15 cells/experiment. Two-way ANOVA; Tukey's multiple comparison test, ****P < 0.0001, **P<0.005.

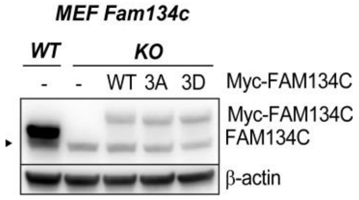

Fig S6. Reconstitution of Fam134c KO MEF with FAM134C mutants. Representative western blot analysis of Fam134c in WT and Fam134c KO MEF cells transfected or not with Myc-FAM134C-WT, -3A or -3D. ß-actin was used as loading control. Arrowhead indicates non-specific band.

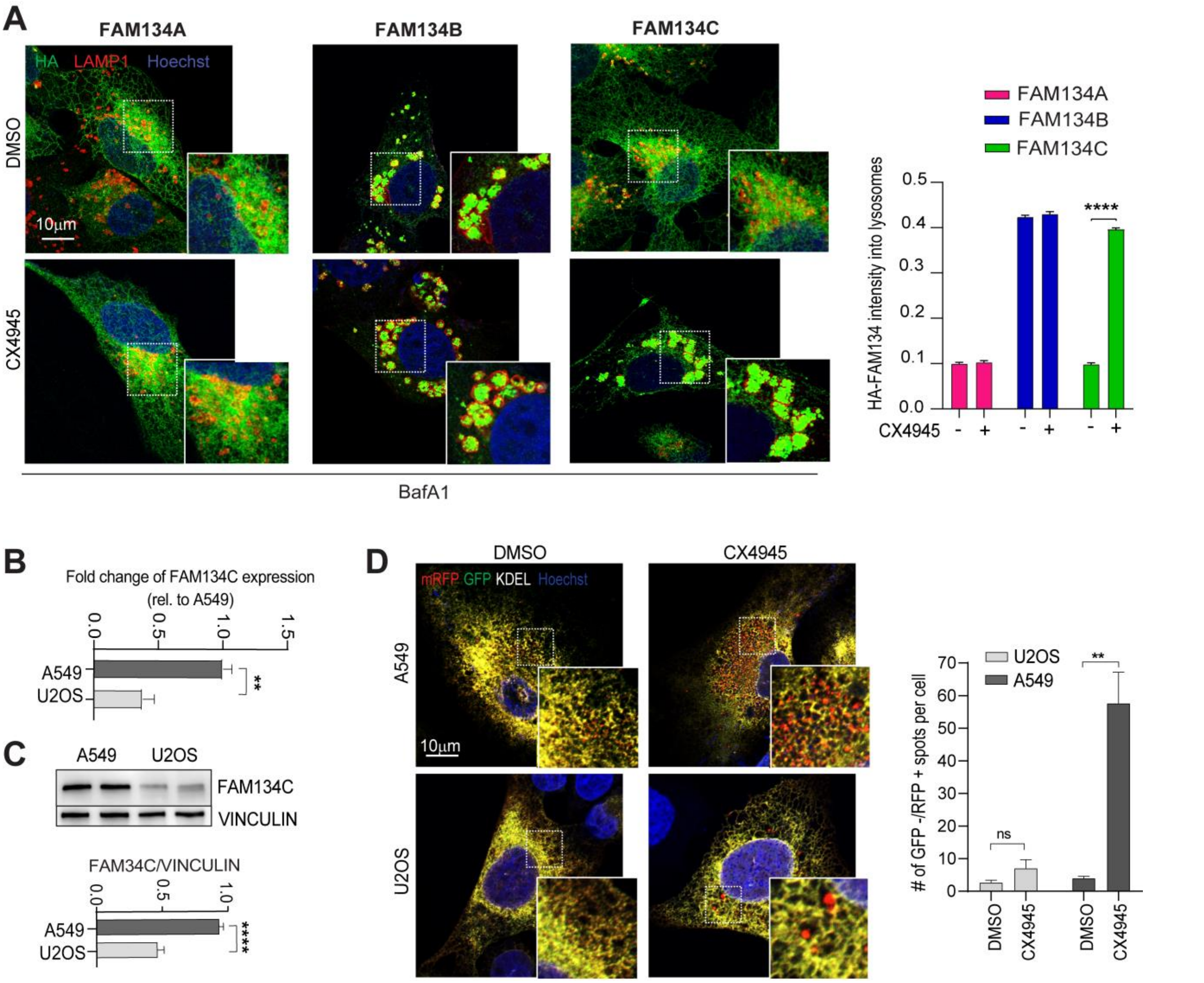

Fig. S7. CK2 inhibition promotes degradation of ER-markers and specifically activates FAM134C. **(A)** Representative images of immunofluorescence staining for HA (green), LAMP1 (red) and nuclei (blue) in U2OS overexpressing doxycycline inducible HA-FAM134-A, -B and -C. Cells were pre-treated for 2h with Bafilomycin (BafA1, 100nM) and then left untreated or treated with CK2 inhibitor CX4945 (6h; 4μM). Scale bar 10μm. On the right, quantification showing the different HA-FAM134s fluorescence intensity in LAMP1 decorated lysosomes. Mean ± SEM. *N*=3 biological replicates. *n*=15 cells/experiment. Two-way ANOVA; Tukey's multiple comparison test, ****P < 0.0001. **(B)** qRT–PCR analysis of *FAM134C* gene expression was analyzed in A549 and U2OS cells. Fold change values were relative to A549 and normalized to HPRT gene. *N*=3 biological replicates. Student's paired t-test **P < 0.005. **(C)** Representative western blot analysis of FAM134C in A549 and U2OS cells. Vinculin was used as loading control. *N*=3 biological replicates. Student's paired t-test, ****P < 0.0001. **(D)** Representative immunofluorescence staining for RFP (red), GFP (green) and nuclei (blue) in A549 and U2OS, stably expressing the ER-phagy reporter ssRFP-GFP-KDEL. Cells were treated with DMSO or CK2 inhibitor CX4945 (10μm, 16h). Scale bar 10μm. On the right, quantification shows the number of red-

only positive puncta (ssRFP+) per cell. Mean ± SEM. N=3 biological replicates. n=15 cells/experiment. Two-way ANOVA; Sidak's test, $^{**}P < 0.005$. ns= not significant.

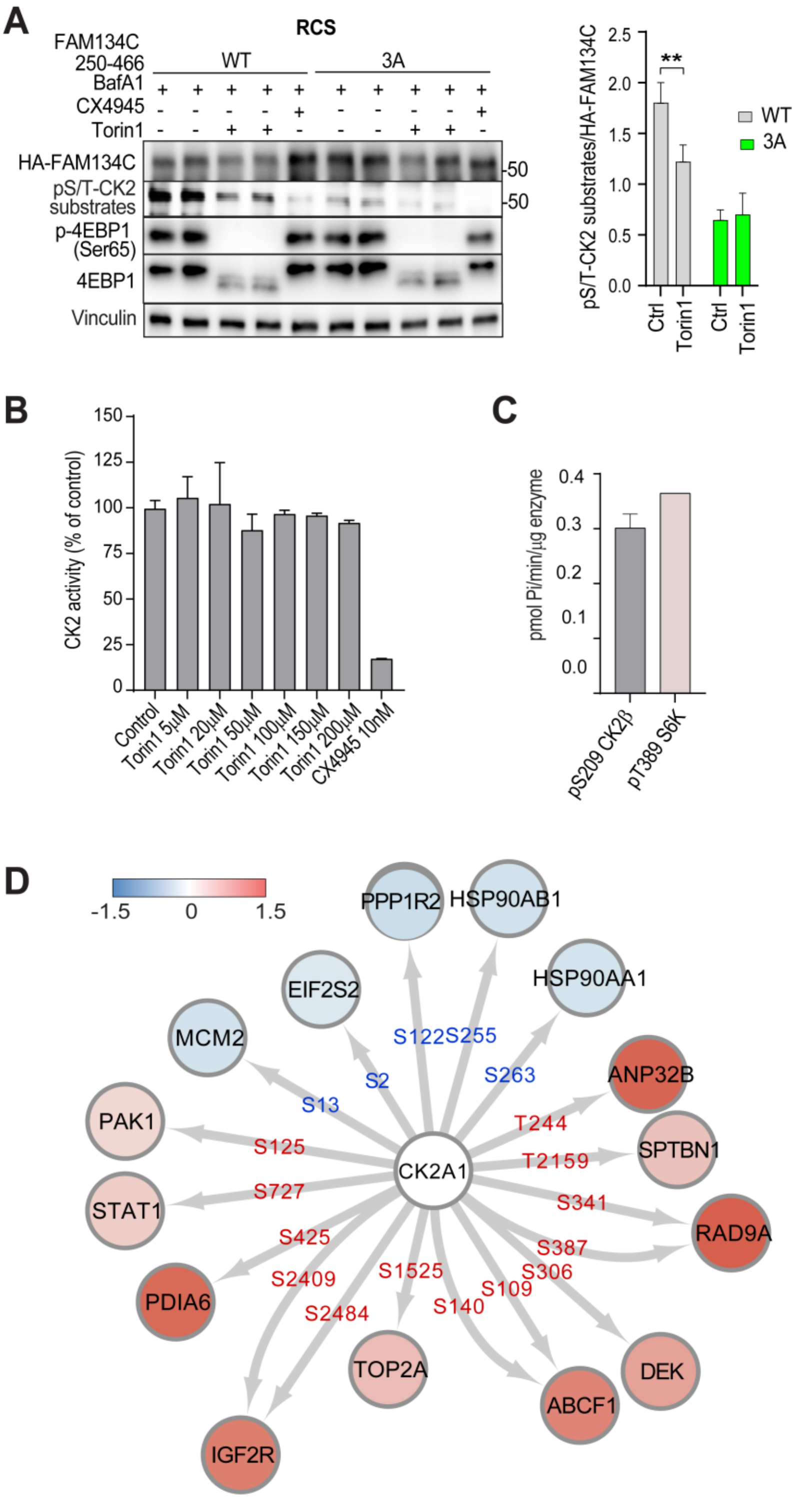

Fig. S8. mTORC1 regulates CK2 substrate selectivity. (A) Western blot analysis of RCS cells transfected with WT- or 3A-HA-FAM134C$^{250\text{-}466}$ peptides and treated with Torin1 (1μM; 6h) and BafA1 (200nM; 6h). Quantification show phosphorylated/total protein ratio. N=5 biological replicates. Mean ± SEM. Two-way ANOVA; Sidak's multiple comparison test, **P<0.005, ***P < 0.0005. **(B)** CK2 activity assay in the presence of increasing concentrations of Torin1. Radioactive activity assays (10 min at 30°C) were performed with recombinant CK2 holoenzyme using the CK2-tide model peptide as substrate and the indicated concentrations of Torin1. 10 nM CX-4945 was used as a control of effective inhibition. Activity is reported as % of the control, in the presence of equal volume of the vehicle. Mean ± SEM,

N=3. **(C)** Radioactive phosphorylation assays using 200 ng mTOR and 1.2 mM of ßCK2 [203-215] peptide or 1.2 mM of S6K[382-393] control peptide for 20 min. Phosphorylation is reported in pmolPi/min/ug enzyme. **(D)** Identification of hypo- and hyper- phosphorylated CK2 substrates at the indicated residues in conditions of starvation (HBSS) *vs* stimulated (amino acids + 100nM Insulin for 20 min) media. *N*=3 biological replicates. FDR < 0.05.

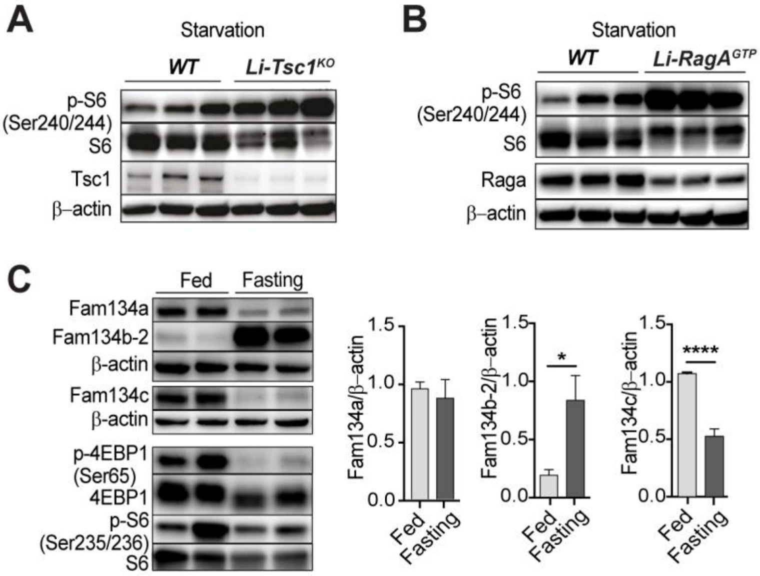

Fig. S9. Liver starvation mainly decreases Fam134c level, among its paralogues. (A-B) Western blot analysis of mTORC1 signalling in liver from WT and *Li-Tsc1*^{KO} (A) and *Li-RagA*^{GTP} (B) Mice fasted for 24h. *N*=3 biological replicates. **(C)** Representative western blot analysis of Fam134 proteins and mTORC1 signalling in liver from fed or starved (overnight) WT mice. β-actin was used as a loading control. *N*=5 (fed), *N*=6 (starved) biological replicates. Bar graph shows data quantification from biological replicates. Student's paired t-test, *P < 0.05, ****P < 0.0001

Supplementary table S1: Table reporting the whole proteome analysis of doxyciclin inducible HA-FAM134s U2OS cells.

Supplementary table S2: Table reporting the whole proteome analysis of FAM134s and Atg7 KO RCS cells.

Supplementary table S3: Table reporting the list of Human Protein Atlas ER-resident and ribosomal proteins.

Supplementary table S4: Table reporting the proteome analysis of doxyciclin inducible HA-FAM134s U2OS cells filtered for ER-resident proteins reported in Supplementary table S3.

Supplementary table S5: Table reporting the proteome analysis of FAM134s and Atg7 KO RCS cells filtered for ER-resident proteins reported in Supplementary table S3.

Supplementary table 6: Table reporting the binding distances of the residues at the interface between FAM134C and LC3B, as extrapolated from the centroid of the most populated cluster family.

Supplementary table 7: Table reporting the Casein kinase 2 (CK2) a and b subunit interaction with FAM134 proteins based on IP-MS interactome experiment from U2OS FLAG-HA-FAM134-A, -B, -C doxycycline inducible cells.

Supplementary table 8: Table reporting all vectors used in this study.

Supplementary table 9: Table reporting all primary antibodies of the present study. Antibodies for Western blot analysis were diluted at 1:1000. Antibodies for immunofluorescence staining were diluted at 1:500.
